# Mechanisms Underlying Target Selectivity for Cell Types and Subcellular Domains in Developing Neocortical Circuits

**DOI:** 10.3389/fncir.2021.728832

**Published:** 2021-09-24

**Authors:** Alan Y. Gutman-Wei, Solange P. Brown

**Affiliations:** ^1^Solomon H. Snyder Department of Neuroscience, Johns Hopkins University School of Medicine, Baltimore, MD, United States; ^2^Kavli Neuroscience Discovery Institute, Johns Hopkins University School of Medicine, Baltimore, MD, United States

**Keywords:** neocortex, pyramidal cell, inhibitory interneuron, synapse formation, development, cell-type specificity, targeting

## Abstract

The cerebral cortex contains numerous neuronal cell types, distinguished by their molecular identity as well as their electrophysiological and morphological properties. Cortical function is reliant on stereotyped patterns of synaptic connectivity and synaptic function among these neuron types, but how these patterns are established during development remains poorly understood. Selective targeting not only of different cell types but also of distinct postsynaptic neuronal domains occurs in many brain circuits and is directed by multiple mechanisms. These mechanisms include the regulation of axonal and dendritic guidance and fine-scale morphogenesis of pre- and postsynaptic processes, lineage relationships, activity dependent mechanisms and intercellular molecular determinants such as transmembrane and secreted molecules, many of which have also been implicated in neurodevelopmental disorders. However, many studies of synaptic targeting have focused on circuits in which neuronal processes target different lamina, such that cell-type-biased connectivity may be confounded with mechanisms of laminar specificity. In the cerebral cortex, each cortical layer contains cell bodies and processes from intermingled neuronal cell types, an arrangement that presents a challenge for the development of target-selective synapse formation. Here, we address progress and future directions in the study of cell-type-biased synaptic targeting in the cerebral cortex. We highlight challenges to identifying developmental mechanisms generating stereotyped patterns of intracortical connectivity, recent developments in uncovering the determinants of synaptic target selection during cortical synapse formation, and current gaps in the understanding of cortical synapse specificity.

## Cell-Type-Biased Connectivity in the Neocortex

The mammalian neocortex is an extensively interconnected network of neurons underlying a broad repertoire of behaviors including perception, decision-making, and voluntary action. Approximately 80–90% of cortical neurons are excitatory while the remaining are inhibitory, and both excitatory and inhibitory neurons are further subdivided into different cell types based on their molecular, morphological and functional characteristics (Kawaguchi and Kubota, 1997; Markram et al., 2004, 2015; Lefort et al., 2009; Meyer et al., 2011; DeFelipe et al., 2013; Greig et al., 2013; Lodato and Arlotta, 2015; Kubota et al., 2016; Tremblay et al., 2016; Baker et al., 2018; Lim et al., 2018a; Tasic et al., 2018; Huang and Paul, 2019; Fishell and Kepecs, 2020; Gouwens et al., 2020; Yuste et al., 2020; Anastasiades and Carter, 2021). The neocortex is traditionally divided into six layers, and each cortical layer is composed of different sets of these neuronal cell types (Callaway, 1998; Thomson and Lamy, 2007; Feldmeyer, 2012; Harris and Mrsic-Flogel, 2013; Harris and Shepherd, 2015; Anastasiades and Carter, 2021; Staiger and Petersen, 2021), most of which also send dendrites and intracortical axons outside their home layer. The function of the mature neocortex relies on stereotyped patterns of intracortical connections among these neuronal cell types both within and across layers as well as predictable properties of their synaptic connections (Thomson and Lamy, 2007; Feldmeyer, 2012; Harris and Shepherd, 2015; Kubota et al., 2016; Tremblay et al., 2016; Adesnik and Naka, 2018; Anastasiades and Carter, 2021; Staiger and Petersen, 2021). Although much progress has been made in identifying mechanisms regulating cell-type-specific synapse formation in other brain areas and model organisms (Kolodkin and Tessier-Lavigne, 2011; Rawson et al., 2017; Favuzzi and Rico, 2018; Apostolo and De Wit, 2019; Kast and Levitt, 2019; Agi et al., 2020; Honig and Shapiro, 2020; Sanes and Zipursky, 2020), how the synaptic organization of intracortical connections arises during development to generate the circuits of the mature mammalian neocortex is only beginning to be understood.

During development, the intracortical axons and dendrites of cortical cell types reach their appropriate laminar destinations (Larsen and Callaway, 2006; Fame et al., 2011; Gibson and Ma, 2011; Kalil and Dent, 2014; Hand et al., 2015; Dorskind and Kolodkin, 2021), and there encounter multiple classes of synaptic targets. They must then select the correct cell types and postsynaptic domains within those cell types with which to form synaptic connections (Thomson and Lamy, 2007; Brown and Hestrin, 2009a; Krook-Magnuson et al., 2012; Harris and Shepherd, 2015; Kubota et al., 2016; Tremblay et al., 2016; Chevée and Brown, 2018; Kast and Levitt, 2019; Anastasiades and Carter, 2021). The appropriate synaptic machinery must also be recruited to the pre- and postsynaptic membranes to establish synaptic properties specific to each connection type (Larsen and Sjöström, 2015; Nusser, 2018; Südhof, 2018; Sanes and Zipursky, 2020). Here, we highlight some of the long-standing barriers to understanding these developmental processes in the neocortex, some mechanisms that have been recently implicated in the development of cell-type-biased intracortical connectivity, and remaining challenges to understanding the cell-type-dependent development of intracortical circuits.

## Peters’ Rule: Axodendritic Overlap Constrains the Development of Intracortical Circuits

**FIGURE 1:**
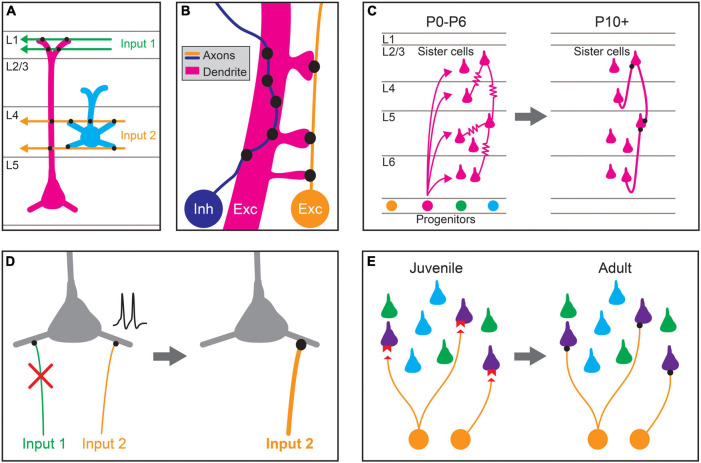
Proposed mechanisms for establishing stereotyped patterns of synaptic connectivity in the neocortex. **(A)** Predictable patterns of synaptic connectivity may be defined by anatomical relationships between presynaptic axons and postsynaptic dendrites. For example, the axodendritic overlap between Input 1 (green) and the two pyramidal cell types (magenta, blue) precludes synapse formation with the blue cell type. The axodendritic overlap of Input 2 (orange) and the two pyramidal cell types predicts a higher probability of connection with the blue type than the magenta type. **(B)** Fine-scale neurite geometry such as axon tortuosity and dendritic spine outgrowth may contribute to synaptic partner selection. Growth patterns of axons and dendrites at small scales alter the amount of apposed membrane between cells, and thus the number of potential synaptic sites. Inhibitory interneuron axons (Inh, blue) in the cortex are highly tortuous, which can increase potential sites of contact with the dendrites of preferred synaptic partners, allowing the formation of more synapses between them. The axons of excitatory pyramidal neurons (Exc, orange) are more linear but directed dendritic spine growth in postsynaptic neurons (Exc, magenta) could also allow for the preferential formation of synaptic sites. **(C)** Lineage relationships affect synaptic connectivity. Clonally related pyramidal cells (sister cells) arising from the same neural progenitor lineage are preferentially interconnected by gap junctions (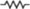) during the first postnatal week (postnatal day 0–6; P0–P6). These gap junctions disappear during the second postnatal week (∼P10+), and chemical synapses are preferentially formed between clonally related pyramidal cells. **(D)** Activity-dependent plasticity may guide cell-type biased synaptic targeting. Different axonal inputs to a cortical cell type may have distinct neural activity patterns, and synapses between specific cell-type combinations may be selectively strengthened (orange input) or weakened (green input) through activity-dependent mechanisms, leading to preferential connectivity between cortical cell types. **(E)** Specific expression of recognition molecules may mediate cell-type or domain-specific synaptic targeting and synapse-type-specific functional properties. During development, preferred synaptic partner cell types may express cognate receptors and ligands (red) on the pre- (orange) and postsynaptic processes (purple). These molecular signals guide growth and synaptogenesis, leading to cell-type-biased connectivity and function in adulthood.

A conceptual barrier to identifying mechanisms that contribute to the development of cell-type dependent synaptic organization in intracortical circuits has been the debate over whether the organization of cortical circuits is fully explained by the dendritic and axonal morphologies of different cell types, or whether additional mechanisms contribute to cell-type-biased connectivity. One hypothesis is that the synaptic connectivity between two cortical cell types reflects their average axodendritic overlap and thus depends only on the densities of the axonal processes of the presynaptic cell types and the dendritic processes of the postsynaptic cell types in the target region (Figure 1A). This concept, known as Peters’ rule, was first posited when describing the connectivity between thalamocortical axons and the postsynaptic elements in layer 4 (L4) of the visual cortex (Peters and Feldman, 1976), and has been extensively applied to studies of cortical connectivity (Braitenberg and Schüz, 1998; Stepanyants and Chklovskii, 2005; Rees et al., 2017). Although Peters’ rule has been extended to different spatial scales (Rees et al., 2017), Peters originally proposed that the synaptic connectivity between cell types reflects the average spatial overlap between the presynaptic axon of one cell type and the different postsynaptic targets in a volume of cortex. If Peters’ rule explains the connectivity patterns of the neocortex, mechanisms governing the development of each cell type’s characteristic axonal and dendritic morphologies, including their vertical and horizontal distribution and their density, would establish the predictable patterns of intracortical synaptic connectivity among cortical cell types by regulating the average axodendritic overlap for different cell-type combinations (Hill et al., 2012; Ramaswamy et al., 2012; Reimann et al., 2017). Additional developmental mechanisms for establishing biases in synaptic connections would not be required. Since synapse formation requires apposition between a presynaptic neuron’s axon and a postsynaptic neuron’s dendrite, by necessity, the synaptic connectivity among different neuron types is constrained by the morphological patterning of their axons and dendrites. However, studies comparing the rate of synaptic connectivity among different cortical cell types with their axodendritic overlap suggest that additional mechanisms must contribute to establishing intracortical circuits.

## Beyond Peters’ Rule: The Challenge of Identifying Additional Cell-Type-Dependent Developmental Mechanisms

Directly testing whether Peters’ rule fully explains stereotyped patterns of intracortical connectivity is technically challenging for several reasons. First, one must know the identity of the cell types in the cortex to compare the synaptic connectivity and morphological relationships for different combinations of cell types. However, despite much progress, a full catalog of the cell types in the neocortex remains out of reach. Indeed, what constitutes a cell type is still debated (Zeng and Sanes, 2017), and increasingly sophisticated methods for determining cell types in the cortex have revealed more and more complexity (Zeisel et al., 2015; Tasic et al., 2016, 2018; Paul et al., 2017; Huang and Paul, 2019; Loo et al., 2019; Gouwens et al., 2020; Kim et al., 2020; Scala et al., 2020; Yao et al., 2020; Yuste et al., 2020). For example, parvalbumin-positive (PV) interneurons, one of the three major classes of cortical inhibitory neurons, have long been further subdivided into two morphological subclasses, basket cells and Chandelier cells (Kawaguchi and Kubota, 1997; Tremblay et al., 2016). However, prior studies using morphological, electrophysiological and molecular criteria as well as recent studies using single cell transcriptomics combined with other characteristics such as cellular morphology have identified multiple types of PV interneurons even within a single cortical layer (DeFelipe et al., 1989; Gupta et al., 2000; Wang et al., 2002; Blatow et al., 2003; Kumar and Ohana, 2008; Tan et al., 2008; Buchanan et al., 2012; Bortone et al., 2014; Jiang et al., 2015; Koelbl et al., 2015; Tasic et al., 2016, 2018; Frandolig et al., 2019; Gouwens et al., 2020; Scala et al., 2020; Yao et al., 2020). These differences have functional consequences. For example, the synapses of deep layer pyramidal neurons onto two types of PV interneurons, one with locally ramifying axons and one that sends axons toward more superficial layers, differ in whether or not they contain presynaptic NMDA receptors (Buchanan et al., 2012). To fully evaluate any selectivity in synaptic connectivity, these different cell types must be taken into account.

In addition to an evolving classification of cortical cell types, methods for comparing the patterns of functional synaptic connectivity with those predicted by neuronal morphology remain low-throughput and technically challenging. Three-dimensional volume electron microscopy reveals the physical connectivity among cells within volumes of cortex, but identifying different cell types within these blocks based on criteria such as long-range axonal targets or transcriptomic classes remains challenging, and the functional properties of the synapses cannot be fully ascertained (Briggman and Bock, 2012; Helmstaedter, 2013; Kubota et al., 2018). Performing paired whole-cell recordings of unitary synaptic connections followed by cell filling and morphological reconstruction is also laborious (Miles and Poncer, 1996; Thomson and Lamy, 2007; Qi et al., 2020). Importantly, these technical limitations have also hindered the ability to test for effects of perturbations of processes hypothesized to underlie the development of intracortical synaptic connections. Despite these obstacles, exceptions to Peters’ rule have been identified, implying the existence of additional mechanisms that contribute to specifying intracortical circuits during development.

## Intracortical Circuits, Synaptic Targeting and Synaptic Specialization

As experimentally measured synaptic connectivity and anatomically predicted connectivity patterns among different cortical cell types have been directly compared, more and more exceptions to connectivity rates based solely on average axodendritic overlap have been observed (Dantzker and Callaway, 2000; Shepherd et al., 2005; Brown and Hestrin, 2009b; Petreanu et al., 2009; Little and Carter, 2012; Kasthuri et al., 2015; Lee et al., 2016; Schmidt et al., 2017; Motta et al., 2019; Karimi et al., 2020). For example, layer 5 corticocortical neurons (L5 CCNs) synapse onto neighboring L5 corticotectal neurons (L5 CTectNs) at a higher rate than predicted from their axodendritic overlap (Brown and Hestrin, 2009b). This general preference for intratelencephalic pyramidal neurons (L5 IT neurons), of which L5 CCNs are a subtype, to synapse onto L5 pyramidal tract neurons (L5 PT neurons), of which L5 CTectNs are a subtype, but not the reverse, has been found across cortical areas (Morishima and Kawaguchi, 2006; Brown and Hestrin, 2009b; Kiritani et al., 2012; Harris and Shepherd, 2015). Similarly, a reconstruction of mouse visual cortex using electron microscopy showed that neurons with similar orientation tuning were preferentially connected even though the axons and dendrites of neurons of all orientation selectivities were intermingled (Lee et al., 2016). Although it remains possible that additional structural constraints contribute to predictable patterns of intracortical connectivity, such as the packing density of neuronal processes of different cortical cell types in sublamina within the cortex (Udvary et al., 2021), these results suggest that mechanisms beyond axodendritic overlap must contribute to preferential synapse formation among some cell types in the cortex.

Synaptic biases for particular neuronal domains have also been shown to be inconsistent with mechanisms for establishing synaptic connections based solely on average axodendritic overlap (Petreanu et al., 2009; Little and Carter, 2012; Reimann et al., 2015; Motta et al., 2019; Schneider-Mizell et al., 2020). For example, different classes of cortical inhibitory neurons target distinct regions of the dendritic and axonal arbors of pyramidal neurons (Jiang et al., 2013; Kubota et al., 2016; Paul et al., 2017), a feature that plays an important role in determining how these inputs are integrated by pyramidal neurons. Chandelier cells (ChCs) form specialized synaptic connections onto the axon initial segments (AISs) of pyramidal neurons (Somogyi, 1977; Fairen and Valverde, 1980; Somogyi et al., 1982; Gallo et al., 2020), in contrast to PV basket cells that preferentially target pyramidal cell somas and proximal dendritic shafts, and somatostatin-expressing (SST) Martinotti cells that synapse onto distal apical dendrites (Kubota, 2014; Kubota et al., 2016; Tremblay et al., 2016; Fishell and Kepecs, 2020). The subcellular preference of neither ChCs (Schneider-Mizell et al., 2020) nor SST Martinotti cells (Reimann et al., 2015) is fully predicted by axodendritic overlap. The developmental targeting of subcellular domains is likely not limited to inhibitory neuron types. For example, the distribution of excitatory and inhibitory synapses around the initial bifurcation of the apical dendritic tuft differs across different classes of pyramidal neurons (Karimi et al., 2020). Similarly, a recent ultrastructural reconstruction of layer 4 (L4) in mouse somatosensory cortex showed that a fraction of both excitatory and inhibitory axons preferentially innervated specific subcellular domains, inconsistent with a purely geometric mechanism (Motta et al., 2019).

Finally, the properties of synaptic connections formed during development can depend on the identity of the pre- or postsynaptic cell type. For example, single pyramidal neurons form both depressing synapses onto PV basket cells and facilitating synapses onto SST Martinotti cells (Reyes et al., 1998; Koester and Johnston, 2005). Similarly, the synapses of deep layer pyramidal neurons onto other pyramidal neurons, SST Martinotti cells and translaminar PV neurons contain presynaptic NMDA receptors while those onto PV basket cells do not (Buchanan et al., 2012). These studies indicate that synaptic machinery must be selectively recruited to particular synapse types to determine connection-type-biased synaptic properties (Nusser, 2018; Südhof, 2018; Sanes and Zipursky, 2020). Together, these results suggest that, although Peters’ rule sets a minimum necessary constraint on synapse formation, additional mechanisms contribute to the predictable synaptic target choices and the development of synaptic properties formed by some cortical cell types during development.

## Cell-Type-Specific Neurite Morphologies Constrain Possible Synaptic Partners

The development of the distinctive intracortical axonal and dendritic morphologies of different cortical cell types, as well as their cell number and position within the cortex, set baseline constraints on the synaptic connectivity between cell types (Figure 1A; Hill et al., 2012; Reimann et al., 2017; Udvary et al., 2021). Mechanisms establishing the characteristic intracortical axonal guidance and branching of different cell types remain incompletely understood but likely include extrinsic molecular cues such as semaphorin, Wnt, netrin, and ephrin family members, interactions with radial glia, selective stabilization of axon collaterals and the cellular migration patterns of inhibitory neurons (Fame et al., 2011; Leyva-Diaz and Lopez-Bendito, 2013; Hand et al., 2015; Dorskind and Kolodkin, 2021). For example, both SST Martinotti cells and translaminar PV interneurons migrate through the marginal zone before arriving into position in the cortex, and preventing this migration impairs the growth of their axonal arbors into the appropriate target layers (Lim et al., 2018b). The size and shape of the dendritic arbors of different classes of cortical neurons are also regulated by multiple mechanisms including extrinsic secreted cues that orient the apical dendrites of pyramidal neurons toward the pial surface (Polleux et al., 2000) as well as intrinsic expression of transcription factors that regulate dendritic arbor complexity and lamination (Chen et al., 2005; Tran et al., 2009; Cubelos et al., 2010, 2015; Lefebvre et al., 2015; Fazel Darbandi et al., 2018). Because the mechanisms regulating axonal and dendritic morphogenesis, together with neuronal positioning and cell number, limit the possible connectivity between cortical cell types during development, they may lead to some predictable patterns of connectivity.

Directed axon growth at finer spatial scales during development (Figure 1B), guided by short-range secreted or contact based cues, may increase the amount of membrane surface apposed to preferred postsynaptic targets, and thus the number of potential synapse sites. The local axonal structure of cortical inhibitory neurons is highly correlated with the dendritic structure of their synaptic target cells, and their axons exhibit high tortuosity (Stepanyants et al., 2004; Portera-Cailliau et al., 2005). This tortuosity suggests that short-range interactions affecting axon outgrowth may generate inhibitory synapse selectivity through increasing membrane contact with target cell types or specific postsynaptic domains, but this hypothesis has not been directly tested. Axon tortuosity may also affect the angular alignment between axons and different dendritic segments, which has been shown to influence the number of synapses formed between axons and target dendrites in the spinal cord (Balaskas et al., 2019). In contrast, the axons of pyramidal neurons are less tortuous and exhibit little spatial correlation with connected neurons (Stepanyants et al., 2004; Kalisman et al., 2005).

The fine-scale structure of dendritic branches and spines may also influence synaptic connectivity with preferred partners (Figure 1B). Not only do the dendritic arbors of cortical pyramidal neurons exceed the theoretical density required for sampling all potential synaptic partners in their dendritic field, they are also studded with dendritic spines that further increase the number of potential sites for synapse formation (Stepanyants et al., 2008; Wen et al., 2009; Bird et al., 2021). Yet pyramids form synapses with only a small subset of available synaptic partners (Song et al., 2005; Stepanyants and Chklovskii, 2005; Kasthuri et al., 2015). Types of nominally aspiny inhibitory interneurons also exhibit spines, albeit at very low densities (Kawaguchi et al., 2006). Spine density and distribution, which varies across cell types and cellular domains, is controlled by a large number of cell-intrinsic and extrinsic factors, including competition between different spine types (Koleske, 2013; Bian et al., 2015; Moyer and Zuo, 2018; Henderson et al., 2019). Directed growth and stabilization of dendritic filopodia has been proposed as a potential strategy for increasing the probability of connection between preferred synaptic partners in the cortex, although direct evidence for such mechanisms has not yet been reported (Dailey and Smith, 1996; Ziv and Smith, 1996; Jontes and Smith, 2000; Bonhoeffer and Yuste, 2002; Konur and Yuste, 2004; Stepanyants et al., 2004; Yuste, 2011).

## Neuronal Lineage Influences Synapse Formation of Clonally Related Neurons

Developmental mechanisms contributing to the pattern of intracortical circuits also reflect the lineage relationships of neurons (Figure 1C). Clonally related excitatory neurons are more likely to be synaptically connected than expected based on their cell types (Yu et al., 2009, 2012; Cadwell et al., 2020), first through the preferential formation of gap junctions among clonally related neurons followed by a transition to chemical synapses (Yu et al., 2009, 2012). Rather than exhibiting increased connectivity within a cell type or cortical layer, these interconnected, clonally related excitatory neurons span multiple cell types and show increased interlaminar connectivity (Cadwell et al., 2020). Formation of these synaptically connected clusters requires normal processes of radial migration and is disrupted by the depletion of DNA-methyltransferase 3 or clustered protocadherins (Tarusawa et al., 2016). Interestingly, clonally related excitatory neurons have similar selectivity for visual stimuli (Li et al., 2012; Ohtsuki et al., 2012). Clonally related inhibitory neurons also preferentially form electrical synapses during development, but do not go on to form preferential chemical synapses (Zhang et al., 2017). Instead, these electrically coupled, clonally related inhibitory neurons tend to target the same set of excitatory neurons (Zhang et al., 2017), but how these synaptic relationships are established is not yet known.

## Activity-Dependent Mechanisms Sculpt Cell-Type-Biased Connectivity

Both spontaneous and evoked neuronal activity play central roles in the development of the neocortex, affecting the number of cells, their position, their intracortical axonal and dendritic morphology and their synaptic connectivity (Katz and Shatz, 1996; Kirischuk et al., 2017; Lim et al., 2018a; Simi and Studer, 2018; Bragg-Gonzalo et al., 2021; Hanganu-Opatz et al., 2021). Activity-dependent strengthening or elimination of specific types of intracortical connections may work in concert with cell-type-specific molecular mechanisms to establish stereotyped patterns of connectivity in cortical circuits (Figure 1D). For example, the preferential connectivity between neurons with similar receptive field properties or activity state suggests that cell-type-biased connectivity is influenced by neuronal activity patterns (Yassin et al., 2010; Ko et al., 2011, 2014; Cossell et al., 2015; Lee et al., 2016). Similarly, while the initial subcellular domain-specific targeting of AISs by ChCs is regulated through molecular mechanisms (Favuzzi et al., 2019; Tai et al., 2019), subsequent changes in neuronal activity modify the location of the AIS and the density of ChC synapses onto AISs (Grubb and Burrone, 2010; Wefelmeyer et al., 2015; Pan-Vazquez et al., 2020). Nonetheless, some stereotyped targeting is maintained despite abnormal cortical activity patterns. For example, the preferential innervation of different subcellular domains by inhibitory neuron subclasses is preserved in organotypic slices (Di Cristo et al., 2004), even though neuronal activity patterns are drastically altered in culture. Although activity-dependent mechanisms likely shape cell-type and domain selectivity of intracortical synaptic connections, the molecular cascades evoked by neuronal activity to affect these processes are still not clear.

## Molecular Mechanisms for Biasing Cell-Type and Domain-Selective Targeting in the Neocortex During Development

In addition to processes regulating axodendritic overlap and directed neurite outgrowth, neural activity, and cell lineage relationships, molecular recognition mechanisms contribute to the stereotyped patterns of connectivity among cortical cell types (Figure 1E). Molecular pathways for establishing cell-type and domain-selective neuronal connections have been well characterized in other model systems and brain areas, including the retina, olfactory bulb, cerebellum, and spinal cord (Sanes and Yamagata, 2009; Shen and Scheiffele, 2010; De Wit and Ghosh, 2016; Rawson et al., 2017; Apostolo and De Wit, 2019; Honig and Shapiro, 2020; Sanes and Zipursky, 2020). Most of these molecules belong to a few families of cell-surface and secreted proteins capable of trans-cellular interactions (Apostolo and De Wit, 2019; Sanes and Zipursky, 2020). The identification of cell-type-biased connectivity in the neocortex suggests that similar mechanisms contribute to cortical development, and recent studies have identified molecular recognition processes that play a role in neocortical synaptic targeting. In the following sections, we first focus on the contributions of molecular mechanisms to domain-specific and cell-type specific synapse formation of inhibitory synapses, including examples where such mechanisms are hypothesized but not yet known (see the sections “Chandelier Cells and Synaptic Targeting at the Axon Initial Segment,” “Somatostatin and Parvalbumin-Expressing Interneurons and Subcellular Targeting,” “Cell-Type-Biased Connections From Inhibitory Interneurons to Pyramidal Neurons,” and “Cell-Type-Specific Inhibitory Networks”). In the subsequent sections (see the sections “Mechanisms Shaping the Synaptic Connectivity of Pyramidal Neurons,” “Mechanisms for Targeting Excitatory Input to Subcellular Domains of Pyramidal Neurons,” and “Cell-Type-Biased Connections From Pyramidal Neuron to Inhibitory Neuron Types”), we describe molecular mechanisms involved in the cell-type and domain-selective targeting of excitatory synapses. Finally, in Section “The Development of Synapse-Type-Specific Functional Properties,” we describe molecular mechanisms implicated in establishing synapse-type-specific functional properties.

### Chandelier Cells and Synaptic Targeting at the Axon Initial Segment

A notable example of subcellular domain targeting by a cortical inhibitory neuron is the ChC, an inhibitory interneuron type that preferentially synapses on the AISs of pyramidal neurons while avoiding somatic and dendritic domains (Somogyi, 1977; Fairen and Valverde, 1980; Somogyi et al., 1982; Gallo et al., 2020). This level of specificity requires additional mechanisms beyond axodendritic overlap (Schneider-Mizell et al., 2020). The preference of ChCs for AISs appears before their specialized axonal cartridges are formed (Gour et al., 2021). Although immature ChCs initially generate some axonal varicosities not associated with AISs, these are subsequently pruned during postnatal development so that by postnatal day 28 (P28) in the mouse, the cells exhibit adult selectivity with the majority of their synapses formed onto AISs (Steinecke et al., 2017; Gour et al., 2021).

**FIGURE 2:**
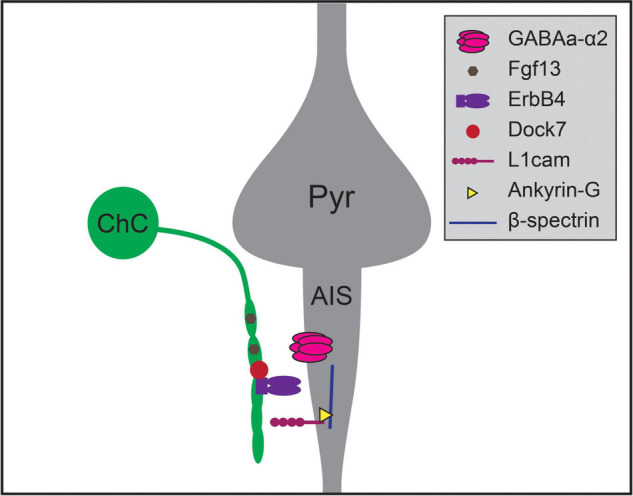
Molecular mechanisms regulating chandelier cell (ChC) targeting of the axon initial segment of pyramidal neurons. Chandelier cells form synapses preferentially on the axon initial segment (AIS) of pyramidal neurons (Pyr), generating specialized axonal structures called cartridges (green). Chandelier cell-AIS targeting requires the expression of Fgf13 and ErbB4 in chandelier cells, and the expression of L1cam and α2 subunit-containing GABA_A_ receptors in the AIS. ErbB4 is further regulated by the expression of intracellular Dock7 in chandelier cells, while L1cam interacts with Ankyrin-G and β-spectrin within the AIS to mediate axo-axonic synaptogenesis.

Recent work has uncovered a number of molecular pathways that underlie ChC synaptic targeting (Figure 2; Contreras et al., 2019; Gallo et al., 2020). One recent study compared results from cell-type-specific RNA sequencing of three classes of developing interneurons – ChC, PV, and SST interneurons – and identified Fgf13, a fibroblast growth factor family member, as required for ChCs to correctly target AISs (Favuzzi et al., 2019). Another recent study found that the interaction of L1cam, a member of the immunoglobulin cell adhesion molecule superfamily, with ankyrin-G at the AIS, is also required to target ChC synapses to AISs (Tai et al., 2019). This mechanism is similar to the process for GABAergic innervation of Purkinje cell AISs directed by the L1cam family member, neurofascin (Ango et al., 2004; Kriebel et al., 2011). Expression of ErbB4, a receptor tyrosine kinase, in ChCs also promotes the formation of axoaxonic synapses, likely through the ErbB4 receptor Neuregulin 1 which is expressed in pyramidal neurons (Fazzari et al., 2010; Del Pino et al., 2013, but see Neddens et al., 2011). ErbB4 is further regulated by the protein Dock7, a member of the DOCK180 family of atypical Rac or Cdc42 GTPase guanine nucleotide exchange factors. Dock7 is required to activate ErbB4 autophosphorylation and promote ChC synaptogenesis (Tai et al., 2014), and may also interact with the α2 subunit of the GABA_A_ receptor which is itself required for synaptogenesis at AISs in the hippocampus (Hines et al., 2018; Yang et al., 2019). Notably, perturbations of most of these mechanisms do not result in complete absence of ChC-AIS targeting, suggesting that multiple molecular mechanisms act together to mediate synaptic specificity.

### Somatostatin and Parvalbumin-Expressing Interneurons and Subcellular Targeting

**FIGURE 3:**
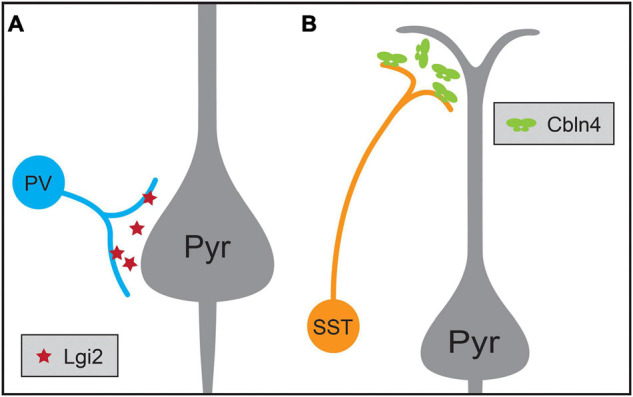
Molecular mechanisms regulating the synaptic targeting of two inhibitory interneuron types to the perisomatic and distal dendritic regions of pyramidal neurons (Pyr), respectively. **(A)** Parvalbumin-positive (PV) interneurons preferentially form synapses onto the perisomatic region of pyramidal neurons, a process that requires the expression of the secreted protein Lgi2 in PV neurons. **(B)** Somatostatin-positive (SST) interneurons preferentially form synapses on the distal dendrites of pyramidal neurons, a process that requires the expression of secreted Cbln4 in SST neurons.

Another example of target selectivity of inhibitory synapses in the neocortex is the biased innervation of different dendritic regions of pyramidal neurons by PV and SST interneurons (Figure 3). Cortical pyramidal neurons are distinguished by their apical dendrite ending in an apical tuft near the pial surface and a domain of basal dendrites surrounding the cell soma. These dendritic domains differ in their integration properties and influence on pyramidal neuron computations (Spruston, 2008; Stuart and Spruston, 2015). SST Martinotti cells and PV interneurons exhibit different preferences for these two dendritic regions: SST Martinotti cells synapse onto the distal apical dendrites of pyramidal neurons while PV interneurons preferentially synapse onto the perisomatic region (Kubota, 2014; Kubota et al., 2016; Tremblay et al., 2016). Comparisons of the transcriptional profiles of developing ChCs, SST and PV interneurons not only identified a role for Fgf13 in directing ChC synapses to the AIS, but also identified molecules contributing to SST and PV targeting of dendritic domains (Favuzzi et al., 2019). Cbln4, a member of the C1q family, is necessary for specifying distal dendrite targeting of SST Martinotti cells and Lgi2, a leucine-rich glioma inactivated family member, for perisomatic targeting of PV basket cells (Figure 3). Furthermore, Cbln4 is sufficient to direct non-SST interneurons to form synapses onto distal dendrites, while not affecting normal targeting of somatic and proximal regions. However, these molecular mechanisms work in concert with additional developmental mechanisms to generate the synaptic patterns seen in the mature cortex: while the synapses of SST interneurons are biased for apical dendrites at the earliest time points tested, those of soma-targeting basket cells also rely on pruning of inappropriate synapses during development (Gour et al., 2021). Similarly, the development of basket cell synapses from cholecystokinin-expressing (CCK) basket cells but not PV interneurons onto pyramidal neurons is regulated by dystroglycan (Fruh et al., 2016; Contreras et al., 2019). These experiments show that interneuron targeting of subcellular domains during development relies on distinct cell-type-specific mechanisms.

### Cell-Type-Biased Connections From Inhibitory Interneurons to Pyramidal Neurons

**FIGURE 4:**
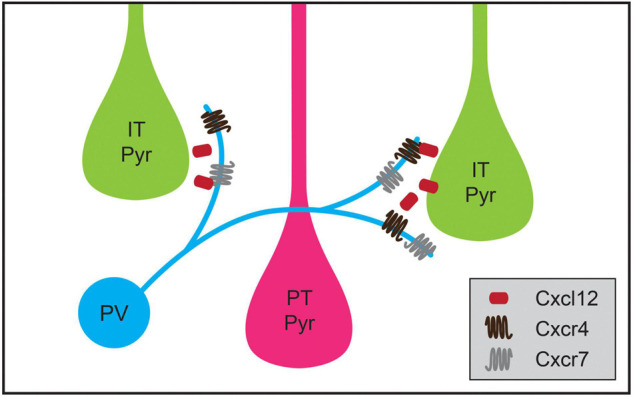
Chemokine signaling regulates cell-type-specific parvalbumin-positive (PV) interneuron targeting in medial prefrontal cortex (mPFC). PV interneurons in mPFC preferentially innervate intratelencephalic pyramidal (IT Pyr) neurons over neighboring pyramidal tract (PT Pyr) neurons. This process is mediated by the selective expression of the secreted protein Cxcl12 at higher levels in IT pyramidal neurons compared to PT pyramidal neurons. Cxcl12 likely acts through its receptors Cxcr4 and Cxcr7, which are expressed in PV interneurons.

Cortical inhibitory interneuron types not only exhibit specificity for a particular cellular region when forming synaptic connections during development but also exhibit cell-type-biased synaptic targeting. As cell types among cortical pyramidal neurons have become better defined, instances of striking specificity in inhibitory targeting of pyramidal neuron subtypes have been identified (Krook-Magnuson et al., 2012; Anastasiades and Carter, 2021). For example, although some studies showed that innervation of pyramidal neurons by PV basket cells and SST Martinotti cells is consistent with Peters’ Rule (Fino and Yuste, 2011; Packer and Yuste, 2011), PV neurons in the mouse medial prefrontal cortex (mPFC) have a higher connection probability with L5 PT cells than IT pyramids (Lee et al., 2014; Anastasiades et al., 2018). Recent work has shown that transsynaptic signaling through chemokine C-X-C motif proteins may play a role in this targeting. In L5 pyramidal neurons, the ligand Cxcl12 is secreted from PT pyramids and helps direct PV neuron axon terminals, which express its receptors Cxcr4 and Cxcr7, to synapse onto perisomatic regions of PT neurons (Figure 4; Wu et al., 2017). Conditional knockout of *Cxcl12* in a subset of L5 pyramidal neurons using the Rbp4-Cre mouse line resulted in a ∼30% decrease in perisomatic inhibitory synapses on L5 pyramidal neurons in mPFC and decreased inhibitory input onto L5 PT but not IT pyramids (Wu et al., 2017). Additional examples of inhibitory neuron types biasing their synaptic output to particular classes of pyramidal neurons have been identified, but the developmental mechanisms establishing these patterns are not yet understood. For example, in layer 2 (L2) of the medial entorhinal cortex, CCK basket cells preferentially innervate one subtype of pyramidal neuron that projects to contralateral entorhinal cortex while avoiding a pyramidal cell subtype that projects to the ipsilateral dentate gyrus (Varga et al., 2010). Similarly, a study using electron microscopy showed that inhibitory axons targeted L1 apical tufts from superficial or deep layer neurons, but not both (Karimi et al., 2020).

Chandelier cells also exhibit cell-type-biased synaptic targeting in addition to selective targeting of AISs. ChCs form axoaxonic synapses onto pyramidal neurons and other ChCs but not onto other interneuron types (Somogyi, 1977; Jiang et al., 2015; Shapson-Coe et al., 2021). Furthermore, ChCs do not synapse onto all the neurons within range of their axonal arbor, and pyramidal cell types receive different numbers of ChC synapses (Somogyi, 1979; Fairen and Valverde, 1980; De Carlos et al., 1985; DeFelipe et al., 1985; Farinas and DeFelipe, 1991; Wang and Sun, 2012; Inan et al., 2013; Lu et al., 2017; Schneider-Mizell et al., 2020). For example, in cat visual cortex, ChCs form more synaptic inputs onto corticocortical neurons whereas corticothalamic neurons receive relatively fewer ChC synapses (Farinas and DeFelipe, 1991). Furthermore, a recent electrophysiological study of connectivity between ChCs and identified pyramidal cell types in prelimbic cortex of mice indicated that L2 ChCs preferentially synapsed onto pyramidal neurons projecting to the basolateral amygdala as compared to those projecting to the contralateral cortex (Lu et al., 2017). Whether differential laminar positioning of pyramidal neurons (Lu et al., 2017; Schneider-Mizell et al., 2020) combined with the distinct morphologies of different types of ChCs (Wang et al., 2019) explains cell-type-specific biases in connectivity must be assessed in concert with testing for molecular mechanisms that may contribute to the formation of these additional levels of selectivity. Nonetheless, these examples suggest that currently unidentified developmental mechanisms sculpt the intracortical connections from inhibitory neurons onto different excitatory cell types.

### Cell-Type-Specific Inhibitory Networks

In addition to forming synapses onto specific neuronal domains and types of cortical excitatory neurons, inhibitory interneurons form cell-type-specific inhibitory networks within the neocortex (Hestrin and Galarreta, 2005; Tremblay et al., 2016; Fishell and Kepecs, 2020; Anastasiades and Carter, 2021). Although the molecular mechanisms underlying the development of these stereotyped patterns of connectivity remain unclear, the increasing availability of genetic tools for identifying and manipulating interneuron subtypes make them potentially tractable systems for investigating molecular recognition mechanisms in neocortical development. For example, PV, SST, and layer 1 (L1) neurogliaform neuron types are each strongly interconnected via electrical synapses (Galarreta and Hestrin, 1999; Gibson et al., 1999; Beierlein et al., 2000; Amitai et al., 2002; Simon et al., 2005). PV interneurons are also interconnected through GABAergic synapses, while rarely innervating other inhibitory neuron subtypes (Galarreta and Hestrin, 1999; Gibson et al., 1999; Pfeffer et al., 2013; Jiang et al., 2015). Studies of inhibitory neuron types have also identified cell-type-biased patterns of connectivity between different inhibitory subtypes (Hestrin and Galarreta, 2005; Tremblay et al., 2016; Fishell and Kepecs, 2020). For example, SST cells are thought to avoid forming chemical synapses onto other SST interneurons while forming GABAergic synapses onto PV interneurons (Gibson et al., 1999; Pfeffer et al., 2013; Xu et al., 2013 but see Jiang et al., 2015), and vasoactive intestinal polypeptide-expressing (VIP) interneurons synapse onto SST Martinotti cells and PV neurons but not onto more numerous neighboring pyramidal neurons (Lee et al., 2013; Pfeffer et al., 2013; Pi et al., 2013). Many additional examples of preferences in synaptic connectivity among inhibitory neuron types indicate that such biases are common in neocortical circuits (Chittajallu et al., 2013; Jiang et al., 2013; Lee et al., 2013, 2015; Pfeffer et al., 2013; Pi et al., 2013; Xu et al., 2013; Kubota et al., 2016; Feldmeyer et al., 2018). As inhibitory neuron types represent only 10–20% of the neurons in the neocortex, these networks represent remarkable stereotyped biases in synaptic targeting. In contrast to interneuron targeting of excitatory cells, molecular mechanisms contributing to the cell-type-specific patterns of electrical and chemical synapses among inhibitory cell types are still unclear.

### Mechanisms Shaping the Synaptic Connectivity of Pyramidal Neurons

The diversity of excitatory cell types in L5 has served as a model for understanding synapse specificity among cortical excitatory neurons. L5 contains two main classes of pyramidal neurons: PT neurons, which project to subcortical brain regions including the spinal cord, brainstem and thalamus, and IT neurons, which confine their axons within the telencephalon, and each may be further subdivided into subtypes (Harris and Shepherd, 2015; Yuste et al., 2020; Anastasiades and Carter, 2021). Studies of mouse sensory cortex showed that the probability of synaptic connectivity reflects the pre- and postsynaptic identity of L5 pyramids and not solely their axodendritic overlap (Brown and Hestrin, 2009b). For example, CCNs, an IT cell class, form more frequent synaptic connections onto CTectNs, a PT cell class, as compared to neighboring CCNs than predicted by their axodendritic overlap (Brown and Hestrin, 2009b). A similar dependence of synaptic connectivity and functional properties on cell type was identified for L5 pyramidal cell types in other cortical areas including motor and frontal cortex (Morishima and Kawaguchi, 2006; Anderson et al., 2010; Morishima et al., 2011; Kiritani et al., 2012). The intracortical connections of L5 neurons thus provide an example of cell-type-biased synaptic targeting among cortical excitatory neurons.

**FIGURE 5:**
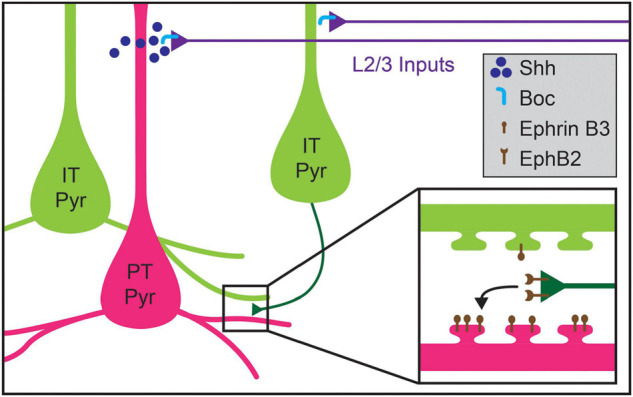
Molecular mechanisms regulating cell-type-specific excitatory connections in cortical layer 5 (L5). Inputs to L5 from layer 2/3 (L2/3) callosal projection neurons are guided by the expression of Shh in pyramidal tract (PT Pyr) neurons (magenta), which signals through its receptor Boc expressed on L2/3 axons (blue). Within L5, intralaminar synapses formed by intratelencephalic pyramidal (IT Pyr) neurons (green) preferentially innervate PT pyramidal neurons over other pyramidal IT neurons. PT pyramidal neurons express higher levels of ephrin B3, which competitively signals through its receptor EphB2 to mediate biased synaptogenesis (inset). This competitive ephrin signaling may underlie the targeting preferences of IT pyramidal neurons.

Recent work has uncovered potential mechanisms for the development of L5 pyramidal neuron connectivity. The formation of the layer 2/3 (2/3) pyramidal projection onto L5 pyramids requires expression of Sonic Hedgehog (*Shh*) by L5 PT neurons and of its receptor, Brother of CDO (*BoC*), in L2/3 axons (Figure 5; Harwell et al., 2012). Perturbing expression of either the receptor or ligand results in decreased L2/3-to-L5 connectivity without affecting connectivity within L2/3 (Harwell et al., 2012). However, whether Shh-Boc signaling alone is sufficient to specify this targeting remains unknown. Furthermore, whether this signaling pathway affects differential innervation of L5 cell types by L2/3 pyramids is also not clear (Otsuka and Kawaguchi, 2008, 2011; Anderson et al., 2010; Collins et al., 2018). Shh may instead establish laminar identity, as suggested by recent work implicating Shh in establishing L5 astrocytic identity (Xie et al., 2020). Competitive signaling between dendritic spines and potential presynaptic partner axons may also play a role in determining cell-type-biased connectivity between L5 pyramidal neurons. Henderson et al. (2019) showed that levels of ephrin B3, a ligand for the Eph family of receptor tyrosine kinases, in postsynaptic spines determine synaptogenesis rates via competitive signaling through EphB2 receptors. Ephrin B3 is significantly enriched in Ctip2+ L5 PT neurons as compared to neighboring Satb2+ L5 IT neurons, providing a potential basis for cell-type-specific synaptic targeting between these cell types (Figure 5; Henderson et al., 2019). New tools, including better genetic access to different L5 cell types, will be required to fully elucidate these mechanisms, including disambiguating layer- and cell-type targeting mechanisms and determining whether competitive expression of signaling molecules in spines underlie such targeting.

### Mechanisms for Targeting Excitatory Input to Subcellular Domains of Pyramidal Neurons

Just as inhibitory neuron subtypes prefer specific subcellular domains of pyramidal neurons, some excitatory inputs are also biased to particular postsynaptic regions. For example, channelrhodopsin-assisted circuit mapping showed that different local and long-range excitatory inputs to L2/3 and L5 pyramidal neurons formed synapses on different regions of their dendritic arbors at relative strengths inconsistent with average axodendritic overlap (Petreanu et al., 2009; Little and Carter, 2012). Similarly, thalamocortical input and the input from different subtypes of L4 excitatory neurons are biased toward different regions of the dendritic arbors of L6 pyramidal neurons (Da Costa and Martin, 2009; Qi and Feldmeyer, 2016). Because the apical dendrites of pyramidal neurons traverse multiple cortical layers, it is possible that developmental mechanisms underlying laminar targeting in the neocortex also contribute to excitatory targeting of subcellular domains, but how these interact with domain-specific mechanisms remains to be tested.

**FIGURE 6:**
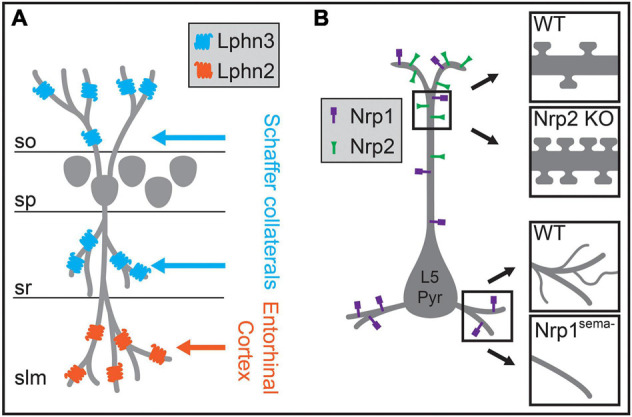
Molecular mechanisms regulating the biased formation of excitatory inputs onto different regions of the postsynaptic dendrite. **(A)** In hippocampal CA1, excitatory Schaffer collateral inputs innervate pyramidal neuron dendrites within the *stratum oriens* (*so*) and the *stratum radiatum* (*sr*), whereas excitatory inputs from the entorhinal cortex form synapses onto distal dendritic regions in the *stratum lacunosum moleculare* (*slm*). In CA1 pyramidal neurons, Lphn3 is localized to *so* and *sr* and is required for Schaffer collateral input targeting to these layers, while Lphn2 is localized to *slm* and is required for targeting of entorhinal cortex inputs to dendrites in *slm*. **(B)** In layer 5 pyramidal (L5 Pyr) neurons in the neocortex, different semaphorin signaling pathways regulate morphology and spine density in distinct dendritic regions. The distribution of the Sema3F receptor Nrp2 is biased to apical dendritic regions, and a null mutation in Nrp2 results in increased spine density on the apical dendrite (upper insets). The Sema3A receptor Nrp1 is localized across the entire dendritic arbor, and a genetic mutation rendering Nrp1 unable to bind Sema3A results in reduced branching complexity of basal dendrites (lower insets). *sp*, stratum pyramidale.

Insights into how these circuits develop in the neocortex may come from developmental processes identified in hippocampal circuits. The hippocampus is a highly laminated structure, with the cell bodies of hippocampal pyramidal neurons contained primarily within a single layer, and their dendrites oriented perpendicularly to layer borders such that each lamina contains dendritic processes of a similar distance from the soma. Excitatory inputs from the entorhinal cortex and from other hippocampal regions segregate into these different laminae, targeting specific dendritic regions of pyramidal neurons (Figure 6A). After being initially directed to the appropriate laminae and regions by interaction with pioneer neurons, guidance molecules, and the expression of topographic partner-matching cues (Skutella and Nitsch, 2001; Förster et al., 2006; Berns et al., 2018), these long-range axons are then directed to the appropriate subcellular domain through domain-specific molecular interactions. Axons originating from each input selectively express binding partners that interact with a diverse complement of transmembrane proteins that are selectively distributed along pyramidal neuron dendrites. For example, in CA1, two closely related G-protein coupled receptors (GPCRs) differentially regulate synapse formation across hippocampal pyramidal neuron dendrites. Lphn2 is enriched in *stratum lacunosum moleculare* and required for the targeting of entorhinal cortex inputs to distal dendrites, while Lphn3 is enriched in both CA1 *stratum radiatum* and *stratum oriens* and is required for the targeting of those layers by Schaffer collateral axons from CA3 and commissural fibers from the contralateral hippocampus (Figure 6A; Anderson et al., 2017; Sando et al., 2019). Subsequent structural studies have shown that this mechanism relies on heterotrimeric transsynaptic binding complexes, and that synapse formation relies on GPCR intracellular signaling (Del Toro et al., 2020; Li et al., 2020b; Sando and Südhof, 2021). These results agree in general with other studies of the hippocampus highlighting the roles of selectively distributed transmembrane molecules in generating laminar or subcellular-domain-specific synaptic targeting. In CA3, interactions between members of the Plexin A family of receptors and the transmembrane semaphorin, Sema6a, restrict mossy fiber axons to the proximal region of pyramidal neuron dendrites (Suto et al., 2007). Similarly, differentially distributed leucine-rich repeat family proteins in CA1 pyramidal neuron dendrites play a role in subcellular targeting by CA3 inputs and play domain-specific roles in controlling synaptic properties (Nishimura-Akiyoshi et al., 2007; DeNardo et al., 2012; Schroeder et al., 2018).

Although the laminar organization of the hippocampus is more precise than in neocortex, the differential distribution of postsynaptic molecules along the dendritic arbors of pyramidal neurons combined with specific expression of binding partners on subsets of presynaptic axons may also play a role in establishing intracortical circuits. Molecular mechanisms that control domain-specific excitatory synaptic density have been discovered in the neocortex (Tran et al., 2009; Cubelos et al., 2015). In L5 pyramidal neurons, expression of the Sema3F receptor Nrp2 is localized to apical dendrites, while the closely related Sema3A receptor Nrp1 is distributed across the dendritic arbor (Figure 6B). Nrp2 controls the density and abundance of spines on apical dendrites, while Nrp1 plays a role in basal dendritic arborization in these neurons (Figure 6B; Gu et al., 2003; Tran et al., 2009). Whether these proteins affect input-type synaptic targeting onto different dendritic regions of L5 pyramidal neurons is still uncertain, but these or related molecular pathways may play a role in excitatory synaptic targeting in the neocortex.

### Cell-Type-Biased Connections From Pyramidal Neuron to Inhibitory Neuron Types

Excitatory cortical pyramidal neurons also selectively target different subtypes of local inhibitory neurons, although molecular recognition processes underlying the development of these circuits are still poorly understood. One study found that L5 IT pyramids that project to contralateral striatum synapsed more frequently onto one particular subtype of low threshold spiking (LTS) interneurons while L5 PT neurons projecting to the pons synapse with similar frequency on all types of L5 LTS interneurons (Morishima et al., 2017). Layer 6 corticothalamic neurons (L6 CThNs) also exhibit biased synaptic targeting of interneurons. L6 CThNs appear to form infrequent or weak synapses onto SST interneurons as well as neighboring excitatory neurons in L6, but target rarer PV interneurons in L6 and L4 via intracortical axon collaterals (Beierlein and Connors, 2002; West et al., 2006; Bortone et al., 2014; Kim et al., 2014; Crandall et al., 2017; Frandolig et al., 2019). The developmental mechanisms that underlie the biases of L5 and L6 pyramids for specific interneuron subtypes remain unclear.

### The Development of Synapse-Type-Specific Functional Properties

**FIGURE 7:**
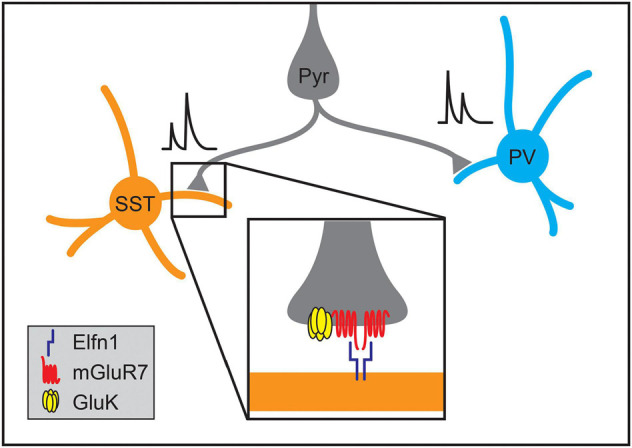
Molecular mechanisms regulating the formation of synapse-type-specific functional properties. The short-term plasticity of pyramidal neuron synapses onto somatostatin-positive (SST) and parvalbumin-positive (PV) interneurons depends on the identity of the postsynaptic cell type. Single pyramidal neurons form facilitating synapses onto SST interneurons while forming depressing synapses onto PV interneurons. The development of facilitation of excitatory synapses onto SST neurons in the hippocampus and neocortex relies on postsynaptic expression of Elfn1 (inset), which then signals through presynaptic metabotropic glutamate receptors (mGluR7). At some synapse types, activation of mGluR7 also interacts with kainate receptors (GluK2) that further enhance synaptic facilitation.

Not only do cell types within the cortex form stereotyped patterns of connectivity, but synapses between different cell types can also acquire distinct signaling characteristics. For example, individual L2/3 pyramidal neurons form depressing synapses onto PV interneurons while forming facilitating synapses onto SST interneurons (Figure 7; Reyes et al., 1998; Koester and Johnston, 2005). The smaller presynaptic calcium transients and facilitating postsynaptic potentials of the synapses onto SST cells suggest lower release probabilities at Pyr➔SST synapses relative to Pyr➔PV connections (Reyes et al., 1998; Koester and Johnston, 2005; Glasgow et al., 2019). Elfn1 has recently been found to regulate the development of synapse-type-specific facilitation in the neocortex and hippocampus, where pyramidal neurons similarly form depressing synapses onto PV neurons and facilitating synapses on SST neurons. In hippocampal CA1, SST neurons in the *stratum oriens* express the transmembrane protein Elfn1, which is localized to excitatory postsynaptic structures and is required to form facilitating synapses (Sylwestrak and Ghosh, 2012). As in CA1, Elfn1 knockout results in the loss of synaptic facilitation at neocortical excitatory synapses on L2/3 and L5 SST neurons (Stachniak et al., 2019) as well as a decrease in facilitation at excitatory connections onto multipolar vasoactive intestinal polypeptide-expressing (VIP) interneurons but not bipolar VIP interneurons (Stachniak et al., 2021). Overexpression of Elfn1 in hippocampal PV neurons is also sufficient to generate facilitating excitatory synapses where depressing synapses would normally occur (Sylwestrak and Ghosh, 2012) as is overexpression in bipolar VIP interneurons (Stachniak et al., 2021).

The mechanisms by which Elfn1 regulates synaptic transmission are being elucidated. Elfn1, localized postsynaptically, binds in *trans* to the metabotropic glutamate receptor, mGluR7, which is localized to presynaptic terminals of Elfn1-positive synapses in both the hippocampus and neocortex (Figure 7; Tomioka et al., 2014; Dunn et al., 2018; Stachniak et al., 2019, 2021). Elfn1 recruits mGluR7 and other group III mGluRs, and can allosterically activate them, modulating their downstream signaling (Dunn et al., 2019). These mechanisms alone or in combination with presynaptic GluK2-containing kainate receptors increase facilitation at different types of synapses in the neocortex and hippocampus (Figure 7; Sylwestrak and Ghosh, 2012; Tomioka et al., 2014; Stachniak et al., 2019, 2021). In VIP interneurons, the transcription factor Prox1 has been implicated in regulating Elfn1 expression (Stachniak et al., 2021).

Many additional examples of synapse-type-specific properties exist in the cortex. Facilitation distinguishes other subsets of cortical synapses, including, for example, thalamocortical (TC) synapses onto SST neurons which facilitate versus TC synapses on PV neurons which depress (Beierlein et al., 2003; Tan et al., 2008). Some pyramidal cell types also form facilitating synapses onto other excitatory cells (Ferster and Lindström, 1985; Stratford et al., 1996; Beierlein and Connors, 2002; Wang et al., 2006; West et al., 2006; Frandolig et al., 2019). The complement of receptors also varies in a synapse-type specific manner. For example, in L5 pyramidal neurons of visual cortex, presynaptic NMDA receptors are specifically localized to synapses made on SST neurons and locally projecting PV neurons, but not on translaminar PV neurons (Buchanan et al., 2012). Similarly, excitatory synapses onto the dendritic shafts and dendritic spines of PV interneurons differ in their enrichment for NMDA receptors (Sancho and Bloodgood, 2018). How these synapse-type-specific properties are established remains unknown.

## Conclusion

Recent studies have begun to reveal developmental mechanisms for biasing the connectivity of cortical cell types and have implicated specific molecules in these processes. However, many challenges to fully understanding these mechanisms remain.

First, a catalog of cell types making up the cortex has not yet been fully established, although an increasingly large repertoire of cortical cell types has been defined using combinations of transcriptional, morphological and electrophysiological data (Zeisel et al., 2015; Tasic et al., 2016, 2018; Paul et al., 2017; Huang and Paul, 2019; Loo et al., 2019; Gouwens et al., 2020; Kim et al., 2020; Scala et al., 2020; Yao et al., 2020; Yuste et al., 2020). Relatedly, although some progress has been made in identifying approaches for directing gene expression in these different cortical cell types, genetic access for many of these is still lacking, preventing manipulation of these cell types during development. Genetic access allows tests of the sufficiency of a developmental mechanism for establishing synaptic connections by, for example, assessing synapse formation with ectopic expression of one or a small number of genes within a pathway, a strong indication that the identified molecules direct synaptic targeting itself.

Second, methods for determining cell-type-biased synaptic connectivity and function remain laborious; thus, not only do the synaptic relationships of cortical cell types remain to be fully elucidated, but it remains difficult to assess how these synaptic relationships change across development or following specific experimental perturbations. Although a recent study successfully used electron microscopy at multiple developmental timepoints to assess the development of intracortical target specificity of different types of inhibitory interneurons (Gour et al., 2021), such studies remain technically difficult.

Third, multiple mechanisms likely work in concert to establish the specificity of intracortical connectivity as is illustrated by ChC targeting of AISs (Figure 2; Gallo et al., 2020), further complicating experimental approaches. Perturbing any single molecule may generate only a subtle phenotype, making it even more difficult to assess any effects.

Fourth, the protein families implicated in synaptic targeting in the neocortex are multifunctional and often affect other aspects of cortical development (De Wit and Ghosh, 2016; Sanes and Zipursky, 2020). Synaptic targeting mechanisms may only function properly in the context of an appropriate temporal sequence of developmental events, requiring tools for temporally specific manipulation such as inducible recombinase systems.

Fifth, most of the work on mechanisms of intracortical synaptic targeting has focused on cell-intrinsic signaling pathways or transcellular interactions between neurons. The roles of additional cortical cell types, like microglia, astrocytes and oligodendrocyte precursor cells, in establishing cell-type-biased connections in the cortex remain underexplored. Glial cells may mediate specific synapse growth or elimination (Schafer and Stevens, 2015; Bosworth and Allen, 2017; Stogsdill and Eroglu, 2017; Buchanan et al., 2021). Furthermore, just as radial glia guide axon growth directions in the cortex, glial cells may act directly to guide cortical synaptic partners to each other, as has been observed for cerebellar Bergmann glia (Ango et al., 2008). More work is required to understand the role glial cells play in cortical synaptic target specificity.

Despite these difficulties, methods such as projection-specific barcoding and profiling of growth cones are being developed that enable unbiased and high-throughput analyses of candidate molecular mechanisms for cortical synaptic targeting (Biesemann et al., 2014; Poulopoulos et al., 2019; Apostolo et al., 2020; Sun et al., 2021). Furthermore, proximity-based labeling methods such as APEX and BioID can be used to specifically tag proteins in the synapses of cell populations of interest, allowing for cell-type and domain-specific profiling of candidate molecules (Loh et al., 2016; Spence et al., 2019; Li et al., 2020a). These new molecular methods provide a toolbox that is particularly useful in the cortex, where the visualization and purification of synapses is difficult amid intermingled cell classes.

Understanding how the intracortical patterns of connectivity are established during development not only has implications for normal cortical development, but also for disease processes. Alterations to the molecular mechanisms of cell-type-biased and synapse-type-specific development may underlie aspects of neurodevelopmental disorders like autism and schizophrenia, as shown by the associations between mutations in *Elfn1* and ASDs, epilepsy, and ADHD (Matsunaga and Aruga, 2021). Similarly, mutations in genes involved in synaptic targeting by ChCs onto the AISs of pyramidal neurons including *L1CAM*, *ERBB4*, and *FGF13* have been implicated in epilepsy, schizophrenia or intellectual disability (Contreras et al., 2019; Gallo et al., 2020). As more mechanisms for specific synaptic targeting are uncovered, it is likely that other links to neurodevelopmental disorders will emerge given the importance of patterned synaptic connectivity to the function of the healthy neocortex (Chen et al., 2014; Nelson and Valakh, 2015).

## Author Contributions

Both authors conceptualized and wrote this review together. Both authors contributed to the article and approved the submitted version.

## Conflict of Interest

The authors declare that the research was conducted in the absence of any commercial or financial relationships that could be construed as a potential conflict of interest.

## Publisher’s Note

All claims expressed in this article are solely those of the authors and do not necessarily represent those of their affiliated organizations, or those of the publisher, the editors and the reviewers. Any product that may be evaluated in this article, or claim that may be made by its manufacturer, is not guaranteed or endorsed by the publisher.

## References

[B1] Adesnik H. Naka A. (2018). Cracking the function of layers in the sensory cortex. *Neuron* 100 1028–1043. 10.1016/j.neuron.2018.10.032 30521778 PMC6342189

[B2] Agi E. Kulkarni A. Hiesinger P. R. (2020). Neuronal strategies for meeting the right partner during brain wiring. *Curr. Opin. Neurobiol.* 63 1–8. 10.1016/j.conb.2020.01.002 32036252 PMC7416442

[B3] Amitai Y. Gibson J. R. Beierlein M. Patrick S. L. Ho A. M. Connors B. W. (2002). The spatial dimensions of electrically coupled networks of interneurons in the neocortex. *J. Neurosci.* 22 4142–4152. 10.1523/JNEUROSCI.22-10-04142.2002 12019332 PMC6757663

[B4] Anastasiades P. G. Carter A. G. (2021). Circuit organization of the rodent medial prefrontal cortex. *Trends Neurosci.* 44 550–563. 10.1016/j.tins.2021.03.006 33972100 PMC8222144

[B5] Anastasiades P. G. Marlin J. J. Carter A. G. (2018). Cell-type specificity of callosally evoked excitation and feedforward inhibition in the prefrontal cortex. *Cell Rep.* 22 679–692. 10.1016/j.celrep.2017.12.073 29346766 PMC5828174

[B6] Anderson C. T. Sheets P. L. Kiritani T. Shepherd G. M. (2010). Sublayer-specific microcircuits of corticospinal and corticostriatal neurons in motor cortex. *Nat. Neurosci.* 13 739–744. 10.1038/nn.2538 20436481 PMC2876193

[B7] Anderson G. R. Maxeiner S. Sando R. Tsetsenis T. Malenka R. C. Südhof T. C. (2017). Postsynaptic adhesion GPCR latrophilin-2 mediates target recognition in entorhinal-hippocampal synapse assembly. *J. Cell Biol.* 216 3831–3846. 10.1083/jcb.201703042 28972101 PMC5674891

[B8] Ango F. Di Cristo G. Higashiyama H. Bennett V. Wu P. Huang Z. J. (2004). Ankyrin-based subcellular gradient of neurofascin, an immunoglobulin family protein, directs GABAergic innervation at purkinje axon initial segment. *Cell* 119 257–272. 10.1016/j.cell.2004.10.004 15479642

[B9] Ango F. Wu C. Van Der Want J. J. Wu P. Schachner M. Huang Z. J. (2008). Bergmann glia and the recognition molecule CHL1 organize GABAergic axons and direct innervation of Purkinje cell dendrites. *PLoS Biol.* 6:e103. 10.1371/journal.pbio.0060103 18447583 PMC2689695

[B10] Apostolo N. De Wit J. (2019). Compartmentalized distributions of neuronal and glial cell-surface proteins pattern the synaptic network. *Curr. Opin. Neurobiol.* 57 126–133. 10.1016/j.conb.2019.01.025 30826628

[B11] Apostolo N. Smukowski S. N. Vanderlinden J. Condomitti G. Rybakin V. Ten Bos J. (2020). Synapse type-specific proteomic dissection identifies IgSF8 as a hippocampal CA3 microcircuit organizer. *Nat. Commun.* 11:5171. 10.1038/s41467-020-18956-x 33057002 PMC7560607

[B12] Baker A. Kalmbach B. Morishima M. Kim J. Juavinett A. Li N. (2018). Specialized subpopulations of deep-layer pyramidal neurons in the neocortex: bridging cellular properties to functional consequences. *J. Neurosci.* 38 5441–5455. 10.1523/JNEUROSCI.0150-18.2018 29798890 PMC6001033

[B13] Balaskas N. Abbott L. F. Jessell T. M. Ng D. (2019). Positional strategies for connection specificity and synaptic organization in spinal sensory-motor circuits. *Neuron* 102 1143–1156.e4. 10.1016/j.neuron.2019.04.008 31076274 PMC7085297

[B14] Beierlein M. Connors B. W. (2002). Short-term dynamics of thalamocortical and intracortical synapses onto layer 6 neurons in neocortex. *J. Neurophysiol.* 88 1924–1932. 10.1152/jn.2002.88.4.1924 12364518

[B15] Beierlein M. Gibson J. R. Connors B. W. (2000). A network of electrically coupled interneurons drives synchronized inhibition in neocortex. *Nat. Neurosci.* 3 904–910. 10.1038/78809 10966621

[B16] Beierlein M. Gibson J. R. Connors B. W. (2003). Two dynamically distinct inhibitory networks in layer 4 of the neocortex. *J. Neurophysiol.* 90 2987–3000. 10.1152/jn.00283.2003 12815025

[B17] Berns D. S. DeNardo L. A. Pederick D. T. Luo L. (2018). Teneurin-3 controls topographic circuit assembly in the hippocampus. *Nature* 554 328–333. 10.1038/nature25463 29414938 PMC7282895

[B18] Bian W. J. Miao W. Y. He S. J. Qiu Z. Yu X. (2015). Coordinated spine pruning and maturation mediated by inter-spine competition for cadherin/catenin complexes. *Cell* 162 808–822. 10.1016/j.cell.2015.07.018 26255771

[B19] Biesemann C. Gronborg M. Luquet E. Wichert S. P. Bernard V. Bungers S. R. (2014). Proteomic screening of glutamatergic mouse brain synaptosomes isolated by fluorescence activated sorting. *EMBO J.* 33 157–170. 10.1002/embj.201386120 24413018 PMC3989609

[B20] Bird A. D. Deters L. H. Cuntz H. (2021). Excess neuronal branching allows for local innervation of specific dendritic compartments in mature cortex. *Cereb. Cortex* 31 1008–1031. 10.1093/cercor/bhaa271 33078188

[B21] Blatow M. Rozov A. Katona I. Hormuzdi S. G. Meyer A. H. Whittington M. A. (2003). A novel network of multipolar bursting interneurons generates theta frequency oscillations in neocortex. *Neuron* 38 805–817. 10.1016/S0896-6273(03)00300-3 12797964

[B22] Bonhoeffer T. Yuste R. (2002). Spine motility: phenomenology, mechanisms, and function. *Neuron* 35 1019–1027. 10.1016/s0896-6273(02)00906-6 12354393

[B23] Bortone D. S. Olsen S. R. Scanziani M. (2014). Translaminar inhibitory cells recruited by layer 6 corticothalamic neurons suppress visual cortex. *Neuron* 82 474–485. 10.1016/j.neuron.2014.02.021 24656931 PMC4068343

[B24] Bosworth A. P. Allen N. J. (2017). The diverse actions of astrocytes during synaptic development. *Curr. Opin. Neurobiol.* 47 38–43. 10.1016/j.conb.2017.08.017 28938161

[B25] Bragg-Gonzalo L. De León Reyes N. S. Nieto M. (2021). Genetic and activity dependent-mechanisms wiring the cortex: two sides of the same coin. *Semin. Cell Dev. Biol.* 10.1016/j.semcdb.2021.05.011 34030948

[B26] Braitenberg V. Schüz A. (1998). *Cortex: Statistics and Geometry of Neuronal Connectivity.* Berlin: Springer-Verlag.

[B27] Briggman K. L. Bock D. D. (2012). Volume electron microscopy for neuronal circuit reconstruction. *Curr. Opin. Neurobiol.* 22 154–161. 10.1016/j.conb.2011.10.022 22119321

[B28] Brown S. P. Hestrin S. (2009a). Cell-type identity: a key to unlocking the function of neocortical circuits. *Curr. Opin. Neurobiol.* 19, 415–421. 10.1016/j.conb.2009.07.011 19674891 PMC2739254

[B29] Brown S. P. Hestrin S. (2009b). Intracortical circuits of pyramidal neurons reflect their long-range axonal targets. *Nature* 457 1133–1136. 10.1038/nature07658 19151698 PMC2727746

[B30] Buchanan J. Elabbady L. Collman F. Jorstad N. L. Bakken T. E. Ott C. (2021). Oligodendrocyte precursor cells prune axons in the mouse neocortex. *bioRxiv* 10.1101/2021.05.29.446047 [Preprint]. PMC9889886 36417438

[B31] Buchanan K. A. Blackman A. V. Moreau A. W. Elgar D. Costa R. P. Lalanne T. (2012). Target-specific expression of presynaptic NMDA receptors in neocortical microcircuits. *Neuron* 75 451–466. 10.1016/j.neuron.2012.06.017 22884329 PMC3657167

[B32] Cadwell C. R. Scala F. Fahey P. G. Kobak D. Mulherkar S. Sinz F. H. (2020). Cell type composition and circuit organization of clonally related excitatory neurons in the juvenile mouse neocortex. *eLife* 9:e52951. 10.7554/eLife.52951 32134385 PMC7162653

[B33] Callaway E. M. (1998). Local circuits in primary visual cortex of the macaque monkey. *Annu. Rev. Neurosci.* 21 47–74. 10.1146/annurev.neuro.21.1.47 9530491

[B34] Chen E. S. Gigek C. O. Rosenfeld J. A. Diallo A. B. Maussion G. Chen G. G. (2014). Molecular convergence of neurodevelopmental disorders. *Am. J. Hum. Genet.* 95 490–508. 10.1016/j.ajhg.2014.09.013 25307298 PMC4225591

[B35] Chen J. G. Rasin M. R. Kwan K. Y. Sestan N. (2005). Zfp312 is required for subcortical axonal projections and dendritic morphology of deep-layer pyramidal neurons of the cerebral cortex. *Proc. Natl. Acad. Sci. U. S. A.* 102 17792–17797. 10.1073/pnas.0509032102 16314561 PMC1308928

[B36] Chevée M. Brown S. P. (2018). The development of local circuits in the neocortex: recent lessons from the mouse visual cortex. *Curr. Opin. Neurobiol.* 53 103–109. 10.1016/j.conb.2018.06.009 30053693 PMC6427921

[B37] Chittajallu R. Pelkey K. A. Mcbain C. J. (2013). Neurogliaform cells dynamically regulate somatosensory integration via synapse-specific modulation. *Nat. Neurosci.* 16 13–15. 10.1038/nn.3284 23222912 PMC4132638

[B38] Collins D. P. Anastasiades P. G. Marlin J. J. Carter A. G. (2018). Reciprocal circuits linking the prefrontal cortex with dorsal and ventral thalamic nuclei. *Neuron* 98 366–379.e4. 10.1016/j.neuron.2018.03.024 29628187 PMC6422177

[B39] Contreras A. Hines D. J. Hines R. M. (2019). Molecular specialization of GABAergic synapses on the soma and axon in cortical and hippocampal circuit function and dysfunction. *Front. Mol. Neurosci.* 12:154. 10.3389/fnmol.2019.00154 31297048 PMC6607995

[B40] Cossell L. Iacaruso M. F. Muir D. R. Houlton R. Sader E. N. Ko H. (2015). Functional organization of excitatory synaptic strength in primary visual cortex. *Nature* 518 399–403. 10.1038/nature14182 25652823 PMC4843963

[B41] Crandall S. R. Patrick S. L. Cruikshank S. J. Connors B. W. (2017). Infrabarrels are layer 6 circuit modules in the barrel cortex that link long-range inputs and outputs. *Cell Rep.* 21 3065–3078. 10.1016/j.celrep.2017.11.049 29241536 PMC5736017

[B42] Cubelos B. Briz C. G. Esteban-Ortega G. M. Nieto M. (2015). Cux1 and Cux2 selectively target basal and apical dendritic compartments of layer II-III cortical neurons. *Dev. Neurobiol.* 75 163–172. 10.1002/dneu.22215 25059644

[B43] Cubelos B. Sebastian-Serrano A. Beccari L. Calcagnotto M. E. Cisneros E. Kim S. (2010). Cux1 and Cux2 regulate dendritic branching, spine morphology, and synapses of the upper layer neurons of the cortex. *Neuron* 66 523–535. 10.1016/j.neuron.2010.04.038 20510857 PMC2894581

[B44] Da Costa N. M. Martin K. A. (2009). Selective targeting of the dendrites of corticothalamic cells by thalamic afferents in area 17 of the cat. *J. Neurosci.* 29 13919–13928. 10.1523/JNEUROSCI.2785-09.2009 19890002 PMC6666723

[B45] Dailey M. E. Smith S. J. (1996). The dynamics of dendritic structure in developing hippocampal slices. *J. Neurosci.* 16 2983–2994. 10.1523/JNEUROSCI.16-09-02983.1996 8622128 PMC6579052

[B46] Dantzker J. L. Callaway E. M. (2000). Laminar sources of synaptic input to cortical inhibitory interneurons and pyramidal neurons. *Nat. Neurosci.* 3 701–707. 10.1038/76656 10862703

[B47] De Carlos J. A. Lopez-Mascaraque L. Valverde F. (1985). Development, morphology and topography of chandelier cells in the auditory cortex of the cat. *Brain Res.* 354 293–300. 10.1016/0165-3806(85)90182-8 4052819

[B48] De Wit J. Ghosh A. (2016). Specification of synaptic connectivity by cell surface interactions. *Nat. Rev. Neurosci.* 17 22–35. 10.1038/nrn.2015.3 26656254

[B49] DeFelipe J. Hendry S. H. Jones E. G. (1989). Visualization of chandelier cell axons by parvalbumin immunoreactivity in monkey cerebral cortex. *Proc. Natl. Acad. Sci. U. S. A.* 86 2093–2097. 10.1073/pnas.86.6.2093 2648389 PMC286854

[B50] DeFelipe J. Hendry S. H. Jones E. G. Schmechel D. (1985). Variability in the terminations of GABAergic chandelier cell axons on initial segments of pyramidal cell axons in the monkey sensory-motor cortex. *J. Comp. Neurol.* 231 364–384. 10.1002/cne.902310307 2981907

[B51] DeFelipe J. Lopez-Cruz P. L. Benavides-Piccione R. Bielza C. Larranaga P. Anderson S. (2013). New insights into the classification and nomenclature of cortical GABAergic interneurons. *Nat. Rev. Neurosci.* 14 202–216. 10.1038/nrn3444 23385869 PMC3619199

[B52] Del Pino I. Garcia-Frigola C. Dehorter N. Brotons-Mas J. R. Alvarez-Salvado E. Martinez De Lagran M. (2013). Erbb4 deletion from fast-spiking interneurons causes schizophrenia-like phenotypes. *Neuron* 79 1152–1168. 10.1016/j.neuron.2013.07.010 24050403

[B53] Del Toro D. Carrasquero-Ordaz M. A. Chu A. Ruff T. Shahin M. Jackson V. A. (2020). Structural basis of teneurin-latrophilin interaction in repulsive guidance of migrating neurons. *Cell* 180 323–339.e19. 10.1016/j.cell.2019.12.014 31928845 PMC6978801

[B54] DeNardo L. A. De Wit J. Otto-Hitt S. Ghosh A. (2012). NGL-2 regulates input-specific synapse development in CA1 pyramidal neurons. *Neuron* 76 762–775. 10.1016/j.neuron.2012.10.013 23177961 PMC7566585

[B55] Di Cristo G. Wu C. Chattopadhyaya B. Ango F. Knott G. Welker E. (2004). Subcellular domain-restricted GABAergic innervation in primary visual cortex in the absence of sensory and thalamic inputs. *Nat. Neurosci.* 7 1184–1186. 10.1038/nn1334 15475951

[B56] Dorskind J. M. Kolodkin A. L. (2021). Revisiting and refining roles of neural guidance cues in circuit assembly. *Curr. Opin. Neurobiol.* 66 10–21. 10.1016/j.conb.2020.07.005 32823181 PMC10725571

[B57] Dunn H. A. Orlandi C. Martemyanov K. A. (2019). Beyond the ligand: extracellular and transcellular G protein-coupled receptor complexes in physiology and pharmacology. *Pharmacol. Rev.* 71 503–519. 10.1124/pr.119.018044 31515243 PMC6742926

[B58] Dunn H. A. Patil D. N. Cao Y. Orlandi C. Martemyanov K. A. (2018). Synaptic adhesion protein ELFN1 is a selective allosteric modulator of group III metabotropic glutamate receptors in trans. *Proc. Natl. Acad. Sci. U. S. A.* 115 5022–5027. 10.1073/pnas.1722498115 29686062 PMC5948991

[B59] Fairen A. Valverde F. (1980). A specialized type of neuron in the visual cortex of cat: a Golgi and electron microscope study of chandelier cells. *J. Comp. Neurol.* 194 761–779. 10.1002/cne.901940405 7204642

[B60] Fame R. M. Macdonald J. L. Macklis J. D. (2011). Development, specification, and diversity of callosal projection neurons. *Trends Neurosci.* 34 41–50. 10.1016/j.tins.2010.10.002 21129791 PMC3053014

[B61] Farinas I. DeFelipe J. (1991). Patterns of synaptic input on corticocortical and corticothalamic cells in the cat visual cortex. II. The axon initial segment. *J. Comp. Neurol.* 304 70–77. 10.1002/cne.903040106 2016413

[B62] Favuzzi E. Deogracias R. Marques-Smith A. Maeso P. Jezequel J. Exposito-Alonso D. (2019). Distinct molecular programs regulate synapse specificity in cortical inhibitory circuits. *Science* 363 413–417. 10.1126/science.aau8977 30679375

[B63] Favuzzi E. Rico B. (2018). Molecular diversity underlying cortical excitatory and inhibitory synapse development. *Curr. Opin. Neurobiol.* 53 8–15. 10.1016/j.conb.2018.03.011 29704699

[B64] Fazel Darbandi S. Robinson Schwartz S. E. Qi Q. Catta-Preta R. Pai E. L. Mandell J. D. (2018). Neonatal Tbr1 dosage controls Cortical layer 6 connectivity. *Neuron* 100 831–845.e7. 10.1016/j.neuron.2018.09.027 30318412 PMC6250594

[B65] Fazzari P. Paternain A. V. Valiente M. Pla R. Lujan R. Lloyd K. (2010). Control of cortical GABA circuitry development by Nrg1 and ErbB4 signalling. *Nature* 464 1376–1380. 10.1038/nature08928 20393464

[B66] Feldmeyer D. (2012). Excitatory neuronal connectivity in the barrel cortex. *Front. Neuroanat.* 6:24. 10.3389/fnana.2012.00024 22798946 PMC3394394

[B67] Feldmeyer D. Qi G. Emmenegger V. Staiger J. F. (2018). Inhibitory interneurons and their circuit motifs in the many layers of the barrel cortex. *Neuroscience* 368 132–151. 10.1016/j.neuroscience.2017.05.027 28528964

[B68] Ferster D. Lindström S. (1985). Augmenting responses evoked in area 17 of the cat by intracortical axon collaterals of cortico-geniculate cells. *J. Physiol.* 367 217–232. 4057097 10.1113/jphysiol.1985.sp015821 PMC1193060

[B69] Fino E. Yuste R. (2011). Dense inhibitory connectivity in neocortex. *Neuron* 69 1188–1203. 10.1016/j.neuron.2011.02.025 21435562 PMC3086675

[B70] Fishell G. Kepecs A. (2020). Interneuron types as attractors and controllers. *Annu. Rev. Neurosci.* 43 1–30. 10.1146/annurev-neuro-070918-050421 31299170 PMC7064158

[B71] Förster E. Zhao S. Frotscher M. (2006). Laminating the hippocampus. *Nat. Rev. Neurosci.* 7 259–267. 10.1038/nrn1882 16543914

[B72] Frandolig J. E. Matney C. J. Lee K. Kim J. Chevée M. Kim S. J. (2019). The synaptic organization of layer 6 circuits reveals inhibition as a major output of a neocortical sublamina. *Cell Rep.* 28 3131–3143.e5. 10.1016/j.celrep.2019.08.048 31533036 PMC6941480

[B73] Fruh S. Romanos J. Panzanelli P. Burgisser D. Tyagarajan S. K. Campbell K. P. (2016). Neuronal dystroglycan is necessary for formation and maintenance of functional CCK-positive basket cell terminals on pyramidal cells. *J. Neurosci.* 36 10296–10313. 10.1523/JNEUROSCI.1823-16.2016 27707967 PMC6705590

[B74] Galarreta M. Hestrin S. (1999). A network of fast-spiking cells in the neocortex connected by electrical synapses. *Nature* 402 72–75. 10.1038/47029 10573418

[B75] Gallo N. B. Paul A. Van Aelst L. (2020). Shedding light on Chandelier cell development, connectivity, and contribution to neural disorders. *Trends Neurosci.* 43 565–580. 10.1016/j.tins.2020.05.003 32564887 PMC7392791

[B76] Gibson D. A. Ma L. (2011). Developmental regulation of axon branching in the vertebrate nervous system. *Development* 138 183–195. 10.1242/dev.046441 21177340 PMC3005597

[B77] Gibson J. R. Beierlein M. Connors B. W. (1999). Two networks of electrically coupled inhibitory neurons in neocortex. *Nature* 402 75–79. 10.1038/47035 10573419

[B78] Glasgow S. D. Mcphedrain R. Madranges J. F. Kennedy T. E. Ruthazer E. S. (2019). Approaches and limitations in the investigation of synaptic transmission and plasticity. *Front. Synaptic Neurosci.* 11:20. 10.3389/fnsyn.2019.00020 31396073 PMC6667546

[B79] Gour A. Boergens K. M. Heike N. Hua Y. Laserstein P. Song K. (2021). Postnatal connectomic development of inhibition in mouse barrel cortex. *Science* 371:eabb4534. 10.1126/science.abb4534 33273061

[B80] Gouwens N. W. Sorensen S. A. Baftizadeh F. Budzillo A. Lee B. R. Jarsky T. (2020). Integrated morphoelectric and transcriptomic classification of cortical GABAergic cells. *Cell* 183 935–953.e19. 10.1016/j.cell.2020.09.057 33186530 PMC7781065

[B81] Greig L. C. Woodworth M. B. Galazo M. J. Padmanabhan H. Macklis J. D. (2013). Molecular logic of neocortical projection neuron specification, development and diversity. *Nat. Rev. Neurosci.* 14 755–769. 10.1038/nrn3586 24105342 PMC3876965

[B82] Grubb M. S. Burrone J. (2010). Activity-dependent relocation of the axon initial segment fine-tunes neuronal excitability. *Nature* 465 1070–1074. 10.1038/nature09160 20543823 PMC3196626

[B83] Gu C. Rodriguez E. R. Reimert D. V. Shu T. Fritzsch B. Richards L. J. (2003). Neuropilin-1 conveys semaphorin and VEGF signaling during neural and cardiovascular development. *Dev. Cell* 5 45–57. 10.1016/s1534-5807(03)00169-2 12852851 PMC3918747

[B84] Gupta A. Wang Y. Markram H. (2000). Organizing principles for a diversity of GABAergic interneurons and synapses in the neocortex. *Science* 287 273–278. 10.1126/science.287.5451.273 10634775

[B85] Hand R. A. Khalid S. Tam E. Kolodkin A. L. (2015). Axon dynamics during neocortical laminar innervation. *Cell Rep.* 12 172–182. 10.1016/j.celrep.2015.06.026 26146079 PMC4517581

[B86] Hanganu-Opatz I. L. Butt S. J. B. Hippenmeyer S. De Marco Garcia N. V. Cardin J. A. Voytek B. (2021). The logic of developing neocortical circuits in health and disease. *J. Neurosci.* 41 813–822. 10.1523/JNEUROSCI.1655-20.2020 33431633 PMC7880298

[B87] Harris K. D. Mrsic-Flogel T. D. (2013). Cortical connectivity and sensory coding. *Nature* 503 51–58. 10.1038/nature12654 24201278

[B88] Harris K. D. Shepherd G. M. (2015). The neocortical circuit: themes and variations. *Nat. Neurosci.* 18 170–181. 10.1038/nn.3917 25622573 PMC4889215

[B89] Harwell C. C. Parker P. R. Gee S. M. Okada A. Mcconnell S. K. Kreitzer A. C. (2012). Sonic hedgehog expression in corticofugal projection neurons directs cortical microcircuit formation. *Neuron* 73 1116–1126. 10.1016/j.neuron.2012.02.009 22445340 PMC3551478

[B90] Helmstaedter M. (2013). Cellular-resolution connectomics: challenges of dense neural circuit reconstruction. *Nat. Methods* 10 501–507. 10.1038/nmeth.2476 23722209

[B91] Henderson N. T. Le Marchand S. J. Hruska M. Hippenmeyer S. Luo L. Dalva M. B. (2019). Ephrin-B3 controls excitatory synapse density through cell-cell competition for EphBs. *eLife* 8:e41563. 10.7554/eLife.41563 30789343 PMC6384025

[B92] Hestrin S. Galarreta M. (2005). Electrical synapses define networks of neocortical GABAergic neurons. *Trends Neurosci.* 28 304–309. 10.1016/j.tins.2005.04.001 15927686

[B93] Hill S. L. Wang Y. Riachi I. Schurmann F. Markram H. (2012). Statistical connectivity provides a sufficient foundation for specific functional connectivity in neocortical neural microcircuits. *Proc. Natl. Acad. Sci. U. S. A.* 109 E2885–E2894. 10.1073/pnas.1202128109 22991468 PMC3479474

[B94] Hines R. M. Maric H. M. Hines D. J. Modgil A. Panzanelli P. Nakamura Y. (2018). Developmental seizures and mortality result from reducing GABAA receptor alpha2-subunit interaction with collybistin. *Nat. Commun.* 9:3130. 10.1038/s41467-018-05481-1 30087324 PMC6081406

[B95] Honig B. Shapiro L. (2020). Adhesion protein structure, molecular affinities, and principles of cell-cell recognition. *Cell* 181 520–535. 10.1016/j.cell.2020.04.010 32359436 PMC7233459

[B96] Huang Z. J. Paul A. (2019). The diversity of GABAergic neurons and neural communication elements. *Nat. Rev. Neurosci.* 20 563–572. 10.1038/s41583-019-0195-4 31222186 PMC8796706

[B97] Inan M. Blazquez-Llorca L. Merchan-Perez A. Anderson S. A. DeFelipe J. Yuste R. (2013). Dense and overlapping innervation of pyramidal neurons by chandelier cells. *J. Neurosci.* 33 1907–1914. 10.1523/JNEUROSCI.4049-12.2013 23365230 PMC3711719

[B98] Jiang X. Shen S. Cadwell C. R. Berens P. Sinz F. Ecker A. S. (2015). Principles of connectivity among morphologically defined cell types in adult neocortex. *Science* 350:aac9462. 10.1126/science.aac9462 26612957 PMC4809866

[B99] Jiang X. Wang G. Lee A. J. Stornetta R. L. Zhu J. J. (2013). The organization of two new cortical interneuronal circuits. *Nat. Neurosci.* 16 210–218. 10.1038/nn.3305 23313910 PMC3589105

[B100] Jontes J. D. Smith S. J. (2000). Filopodia, spines, and the generation of synaptic diversity. *Neuron* 27 11–14. 10.1016/s0896-6273(00)00003-9 10939326

[B101] Kalil K. Dent E. W. (2014). Branch management: mechanisms of axon branching in the developing vertebrate CNS. *Nat. Rev. Neurosci.* 15 7–18. 10.1038/nrn3650 24356070 PMC4063290

[B102] Kalisman N. Silberberg G. Markram H. (2005). The neocortical microcircuit as a tabula rasa. *Proc. Natl. Acad. Sci. U. S. A.* 102 880–885. 10.1073/pnas.0407088102 15630093 PMC545526

[B103] Karimi A. Odenthal J. Drawitsch F. Boergens K. M. Helmstaedter M. (2020). Cell-type specific innervation of cortical pyramidal cells at their apical dendrites. *eLife* 9:e46876. 10.7554/eLife.46876 32108571 PMC7297530

[B104] Kast R. J. Levitt P. (2019). Precision in the development of neocortical architecture: from progenitors to cortical networks. *Prog. Neurobiol.* 175 77–95. 10.1016/j.pneurobio.2019.01.003 30677429 PMC6402587

[B105] Kasthuri N. Hayworth K. J. Berger D. R. Schalek R. L. Conchello J. A. Knowles-Barley S. (2015). Saturated reconstruction of a volume of neocortex. *Cell* 162 648–661. 10.1016/j.cell.2015.06.054 26232230

[B106] Katz L. C. Shatz C. J. (1996). Synaptic activity and the construction of cortical circuits. *Science* 274 1133–1138. 10.1126/science.274.5290.1133 8895456

[B107] Kawaguchi Y. Karube F. Kubota Y. (2006). Dendritic branch typing and spine expression patterns in cortical nonpyramidal cells. *Cereb. Cortex* 16 696–711. 10.1093/cercor/bhj015 16107588

[B108] Kawaguchi Y. Kubota Y. (1997). GABAergic cell subtypes and their synaptic connections in rat frontal cortex. *Cereb. Cortex* 7 476–486. 10.1093/cercor/7.6.476 9276173

[B109] Kim E. J. Zhang Z. Huang L. Ito-Cole T. Jacobs M. W. Juavinett A. L. (2020). Extraction of distinct neuronal cell types from within a genetically continuous population. *Neuron* 107 274–282.e6. 10.1016/j.neuron.2020.04.018 32396852 PMC7381365

[B110] Kim J. Matney C. J. Blankenship A. Hestrin S. Brown S. P. (2014). Layer 6 corticothalamic neurons activate a cortical output layer, layer 5a. *J. Neurosci.* 34 9656–9664. 10.1523/JNEUROSCI.1325-14.2014 25031405 PMC4099543

[B111] Kirischuk S. Sinning A. Blanquie O. Yang J. W. Luhmann H. J. Kilb W. (2017). Modulation of neocortical development by early neuronal activity: physiology and pathophysiology. *Front. Cell Neurosci.* 11:379. 10.3389/fncel.2017.00379 29238291 PMC5712676

[B112] Kiritani T. Wickersham I. R. Seung H. S. Shepherd G. M. (2012). Hierarchical connectivity and connection-specific dynamics in the corticospinal-corticostriatal microcircuit in mouse motor cortex. *J. Neurosci.* 32 4992–5001. 10.1523/JNEUROSCI.4759-11.2012 22492054 PMC3329752

[B113] Ko H. Hofer S. B. Pichler B. Buchanan K. A. Sjöström P. J. Mrsic-Flogel T. D. (2011). Functional specificity of local synaptic connections in neocortical networks. *Nature* 473 87–91. 10.1038/nature09880 21478872 PMC3089591

[B114] Ko H. Mrsic-Flogel T. D. Hofer S. B. (2014). Emergence of feature-specific connectivity in cortical microcircuits in the absence of visual experience. *J. Neurosci.* 34 9812–9816. 10.1523/JNEUROSCI.0875-14.2014 25031418 PMC4099553

[B115] Koelbl C. Helmstaedter M. Lubke J. Feldmeyer D. (2015). A barrel-related interneuron in layer 4 of rat somatosensory cortex with a high intrabarrel connectivity. *Cereb. Cortex* 25 713–725. 10.1093/cercor/bht263 24076498 PMC4318534

[B116] Koester H. J. Johnston D. (2005). Target cell-dependent normalization of transmitter release at neocortical synapses. *Science* 308 863–866. 10.1126/science.1100815 15774725

[B117] Koleske A. J. (2013). Molecular mechanisms of dendrite stability. *Nat. Rev. Neurosci.* 14 536–550. 10.1038/nrn3486 23839597 PMC3947514

[B118] Kolodkin A. L. Tessier-Lavigne M. (2011). Mechanisms and molecules of neuronal wiring: a primer. *Cold Spring Harb. Perspect. Biol.* 3:a001727 10.1101/cshperspect.a001727 21123392 PMC3098670

[B119] Konur S. Yuste R. (2004). Imaging the motility of dendritic protrusions and axon terminals: roles in axon sampling and synaptic competition. *Mol. Cell. Neurosci.* 27 427–440. 10.1016/j.mcn.2004.07.005 15555921

[B120] Kriebel M. Metzger J. Trinks S. Chugh D. Harvey R. J. Harvey K. (2011). The cell adhesion molecule neurofascin stabilizes axo-axonic GABAergic terminals at the axon initial segment. *J. Biol. Chem.* 286 24385–24393. 10.1074/jbc.M110.212191 21576239 PMC3129217

[B121] Krook-Magnuson E. Varga C. Lee S. H. Soltesz I. (2012). New dimensions of interneuronal specialization unmasked by principal cell heterogeneity. *Trends Neurosci.* 35 175–184. 10.1016/j.tins.2011.10.005 22119146 PMC3294038

[B122] Kubota Y. (2014). Untangling GABAergic wiring in the cortical microcircuit. *Curr. Opin. Neurobiol.* 26 7–14. 10.1016/j.conb.2013.10.003 24650498

[B123] Kubota Y. Karube F. Nomura M. Kawaguchi Y. (2016). The diversity of cortical inhibitory synapses. *Front. Neural. Circuits* 10:27. 10.3389/fncir.2016.00027 27199670 PMC4842771

[B124] Kubota Y. Sohn J. Kawaguchi Y. (2018). Large volume electron microscopy and neural microcircuit analysis. *Front. Neural. Circuits* 12:98. 10.3389/fncir.2018.00098 30483066 PMC6240581

[B125] Kumar P. Ohana O. (2008). Inter- and intralaminar subcircuits of excitatory and inhibitory neurons in layer 6a of the rat barrel cortex. *J. Neurophysiol.* 100 1909–1922. 10.1152/jn.90684.2008 18650305

[B126] Larsen D. D. Callaway E. M. (2006). Development of layer-specific axonal arborizations in mouse primary somatosensory cortex. *J. Comp. Neurol.* 494 398–414. 10.1002/cne.20754 16320250 PMC4651208

[B127] Larsen R. S. Sjöström P. J. (2015). Synapse-type-specific plasticity in local circuits. *Curr. Opin. Neurobiol.* 35 127–135. 10.1016/j.conb.2015.08.001 26310110 PMC5280068

[B128] Lee A. J. Wang G. Jiang X. Johnson S. M. Hoang E. T. Lante F. (2015). Canonical organization of layer 1 neuron-led cortical inhibitory and disinhibitory interneuronal circuits. *Cereb. Cortex* 25 2114–2126. 10.1093/cercor/bhu020 24554728 PMC4494026

[B129] Lee A. T. Gee S. M. Vogt D. Patel T. Rubenstein J. L. Sohal V. S. (2014). Pyramidal neurons in prefrontal cortex receive subtype-specific forms of excitation and inhibition. *Neuron* 81 61–68. 10.1016/j.neuron.2013.10.031 24361076 PMC3947199

[B130] Lee S. Kruglikov I. Huang Z. J. Fishell G. Rudy B. (2013). A disinhibitory circuit mediates motor integration in the somatosensory cortex. *Nat. Neurosci.* 16 1662–1670. 10.1038/nn.3544 24097044 PMC4100076

[B131] Lee W. C. Bonin V. Reed M. Graham B. J. Hood G. Glattfelder K. (2016). Anatomy and function of an excitatory network in the visual cortex. *Nature* 532 370–374. 10.1038/nature17192 27018655 PMC4844839

[B132] Lefebvre J. L. Sanes J. R. Kay J. N. (2015). Development of dendritic form and function. *Annu. Rev. Cell Dev. Biol.* 31 741–777. 10.1146/annurev-cellbio-100913-013020 26422333

[B133] Lefort S. Tomm C. Floyd Sarria J. C. Petersen C. C. (2009). The excitatory neuronal network of the C2 barrel column in mouse primary somatosensory cortex. *Neuron* 61 301–316. 10.1016/j.neuron.2008.12.020 19186171

[B134] Leyva-Diaz E. Lopez-Bendito G. (2013). In and out from the cortex: development of major forebrain connections. *Neuroscience* 254 26–44. 10.1016/j.neuroscience.2013.08.070 24042037

[B135] Li J. Han S. Li H. Udeshi N. D. Svinkina T. Mani D. R. (2020a). Cell-surface proteomic profiling in the fly brain uncovers wiring regulators. *Cell* 180 373–386.e15. 10.1016/j.cell.2019.12.029 31955847 PMC7072036

[B136] Li J. Xie Y. Cornelius S. Jiang X. Sando R. Kordon S. P. (2020b). Alternative splicing controls teneurin-latrophilin interaction and synapse specificity by a shape-shifting mechanism. *Nat. Commun.* 11:2140. 10.1038/s41467-020-16029-7 32358586 PMC7195488

[B137] Li Y. Lu H. Cheng P. L. Ge S. Xu H. Shi S. H. (2012). Clonally related visual cortical neurons show similar stimulus feature selectivity. *Nature* 486 118–121. 10.1038/nature11110 22678292 PMC3375857

[B138] Lim L. Mi D. Llorca A. Marin O. (2018a). Development and functional diversification of cortical interneurons. *Neuron* 100 294–313. 10.1016/j.neuron.2018.10.009 30359598 PMC6290988

[B139] Lim L. Pakan J. M. P. Selten M. M. Marques-Smith A. Llorca A. Bae S. E. (2018b). Optimization of interneuron function by direct coupling of cell migration and axonal targeting. *Nat. Neurosci.* 21 920–931. 10.1038/s41593-018-0162-9 29915195 PMC6061935

[B140] Little J. P. Carter A. G. (2012). Subcellular synaptic connectivity of layer 2 pyramidal neurons in the medial prefrontal cortex. *J. Neurosci.* 32 12808–12819. 10.1523/JNEUROSCI.1616-12.2012 22973004 PMC3490687

[B141] Lodato S. Arlotta P. (2015). Generating neuronal diversity in the mammalian cerebral cortex. *Annu. Rev. Cell Dev. Biol.* 31 699–720. 10.1146/annurev-cellbio-100814-125353 26359774 PMC4778709

[B142] Loh K. H. Stawski P. S. Draycott A. S. Udeshi N. D. Lehrman E. K. Wilton D. K. (2016). Proteomic analysis of unbounded cellular compartments: synaptic clefts. *Cell* 166 1295–1307.e21. 10.1016/j.cell.2016.07.041 27565350 PMC5167540

[B143] Loo L. Simon J. M. Xing L. Mccoy E. S. Niehaus J. K. Guo J. (2019). Single-cell transcriptomic analysis of mouse neocortical development. *Nat. Commun.* 10:134. 10.1038/s41467-018-08079-9 30635555 PMC6329831

[B144] Lu J. Tucciarone J. Padilla-Coreano N. He M. Gordon J. A. Huang Z. J. (2017). Selective inhibitory control of pyramidal neuron ensembles and cortical subnetworks by chandelier cells. *Nat. Neurosci.* 20 1377–1383. 10.1038/nn.4624 28825718 PMC5614838

[B145] Markram H. Muller E. Ramaswamy S. Reimann M. W. Abdellah M. Sanchez C. A. (2015). Reconstruction and simulation of neocortical microcircuitry. *Cell* 163 456–492. 10.1016/j.cell.2015.09.029 26451489

[B146] Markram H. Toledo-Rodriguez M. Wang Y. Gupta A. Silberberg G. Wu C. (2004). Interneurons of the neocortical inhibitory system. *Nat. Rev. Neurosci.* 5 793–807. 10.1038/nrn1519 15378039

[B147] Matsunaga H. Aruga J. (2021). Trans-synaptic regulation of metabotropic glutamate receptors by Elfn proteins in health and disease. *Front. Neural. Circuits* 15:634875. 10.3389/fncir.2021.634875 33790745 PMC8005653

[B148] Meyer H. S. Schwarz D. Wimmer V. C. Schmitt A. C. Kerr J. N. Sakmann B. (2011). Inhibitory interneurons in a cortical column form hot zones of inhibition in layers 2 and 5A. *Proc. Natl. Acad. Sci. U. S. A.* 108 16807–16812. 10.1073/pnas.1113648108 21949377 PMC3189020

[B149] Miles R. Poncer J. C. (1996). Paired recordings from neurones. *Curr. Opin. Neurobiol.* 6 387–394. 10.1016/s0959-4388(96)80124-3 8794081

[B150] Morishima M. Kawaguchi Y. (2006). Recurrent connection patterns of corticostriatal pyramidal cells in frontal cortex. *J. Neurosci.* 26 4394–4405. 10.1523/JNEUROSCI.0252-06.2006 16624959 PMC6674016

[B151] Morishima M. Kobayashi K. Kato S. Kawaguchi Y. (2017). Segregated excitatory-inhibitory recurrent subnetworks in Layer 5 of the rat frontal cortex. *Cereb. Cortex* 27 5846–5857. 10.1093/cercor/bhx276 29045559 PMC5905586

[B152] Morishima M. Morita K. Kubota Y. Kawaguchi Y. (2011). Highly differentiated projection-specific cortical subnetworks. *J. Neurosci.* 31 10380–10391. 10.1523/JNEUROSCI.0772-11.2011 21753015 PMC6623049

[B153] Motta A. Berning M. Boergens K. M. Staffler B. Beining M. Loomba S. (2019). Dense connectomic reconstruction in layer 4 of the somatosensory cortex. *Science* 366:eaay3134. 10.1126/science.aay3134 31649140

[B154] Moyer C. E. Zuo Y. (2018). Cortical dendritic spine development and plasticity: insights from in vivo imaging. *Curr. Opin. Neurobiol.* 53 76–82. 10.1016/j.conb.2018.06.002 29936406

[B155] Neddens J. Fish K. N. Tricoire L. Vullhorst D. Shamir A. Chung W. (2011). Conserved interneuron-specific ErbB4 expression in frontal cortex of rodents, monkeys, and humans: implications for schizophrenia. *Biol. Psychiatry* 70 636–645. 10.1016/j.biopsych.2011.04.016 21664604 PMC5040357

[B156] Nelson S. B. Valakh V. (2015). Excitatory/inhibitory balance and circuit homeostasis in autism spectrum disorders. *Neuron* 87 684–698. 10.1016/j.neuron.2015.07.033 26291155 PMC4567857

[B157] Nishimura-Akiyoshi S. Niimi K. Nakashiba T. Itohara S. (2007). Axonal netrin-Gs transneuronally determine lamina-specific subdendritic segments. *Proc. Natl. Acad. Sci. U. S. A.* 104 14801–14806. 10.1073/pnas.0706919104 17785411 PMC1964543

[B158] Nusser Z. (2018). Creating diverse synapses from the same molecules. *Curr. Opin. Neurobiol.* 51 8–15. 10.1016/j.conb.2018.01.001 29353084

[B159] Ohtsuki G. Nishiyama M. Yoshida T. Murakami T. Histed M. Lois C. (2012). Similarity of visual selectivity among clonally related neurons in visual cortex. *Neuron* 75 65–72. 10.1016/j.neuron.2012.05.023 22794261

[B160] Otsuka T. Kawaguchi Y. (2008). Firing-pattern-dependent specificity of cortical excitatory feed-forward subnetworks. *J. Neurosci.* 28 11186–11195. 10.1523/JNEUROSCI.1921-08.2008 18971461 PMC6671518

[B161] Otsuka T. Kawaguchi Y. (2011). Cell diversity and connection specificity between callosal projection neurons in the frontal cortex. *J. Neurosci.* 31 3862–3870. 10.1523/JNEUROSCI.5795-10.2011 21389241 PMC6622807

[B162] Packer A. M. Yuste R. (2011). Dense, unspecific connectivity of neocortical parvalbumin-positive interneurons: a canonical microcircuit for inhibition? *J. Neurosci.* 31 13260–13271. 10.1523/JNEUROSCI.3131-11.2011 21917809 PMC3178964

[B163] Pan-Vazquez A. Wefelmeyer W. Gonzalez Sabater V. Neves G. Burrone J. (2020). Activity-dependent plasticity of axo-axonic synapses at the axon initial segment. *Neuron* 106 265–276.e6. 10.1016/j.neuron.2020.01.037 32109363 PMC7181187

[B164] Paul A. Crow M. Raudales R. He M. Gillis J. Huang Z. J. (2017). Transcriptional architecture of synaptic communication delineates GABAergic neuron identity. *Cell* 171 522–539.e20. 10.1016/j.cell.2017.08.032 28942923 PMC5772785

[B165] Peters A. Feldman M. L. (1976). The projection of the lateral geniculate nucleus to area 17 of the rat cerebral cortex. I. General description. *J. Neurocytol.* 5 63–84. 10.1007/BF01176183 1249593

[B166] Petreanu L. Mao T. Sternson S. M. Svoboda K. (2009). The subcellular organization of neocortical excitatory connections. *Nature* 457 1142–1145. 10.1038/nature07709 19151697 PMC2745650

[B167] Pfeffer C. K. Xue M. He M. Huang Z. J. Scanziani M. (2013). Inhibition of inhibition in visual cortex: the logic of connections between molecularly distinct interneurons. *Nat. Neurosci.* 16 1068–1076. 10.1038/nn.3446 23817549 PMC3729586

[B168] Pi H. J. Hangya B. Kvitsiani D. Sanders J. I. Huang Z. J. Kepecs A. (2013). Cortical interneurons that specialize in disinhibitory control. *Nature* 503 521–524. 10.1038/nature12676 24097352 PMC4017628

[B169] Polleux F. Morrow T. Ghosh A. (2000). Semaphorin 3A is a chemoattractant for cortical apical dendrites. *Nature* 404 567–573. 10.1038/35007001 10766232

[B170] Portera-Cailliau C. Weimer R. M. De Paola V. Caroni P. Svoboda K. (2005). Diverse modes of axon elaboration in the developing neocortex. *PLoS Biol.* 3:e272. 10.1371/journal.pbio.0030272 16026180 PMC1180514

[B171] Poulopoulos A. Murphy A. J. Ozkan A. Davis P. Hatch J. Kirchner R. (2019). Subcellular transcriptomes and proteomes of developing axon projections in the cerebral cortex. *Nature* 565 356–360. 10.1038/s41586-018-0847-y 30626971 PMC6484835

[B172] Qi G. Feldmeyer D. (2016). Dendritic target region-specific formation of synapses between excitatory layer 4 neurons and layer 6 pyramidal cells. *Cereb. Cortex* 26 1569–1579. 10.1093/cercor/bhu334 25595180

[B173] Qi G. Yang D. Ding C. Feldmeyer D. (2020). Unveiling the synaptic function and structure using paired recordings from synaptically coupled neurons. *Front. Synaptic Neurosci.* 12:5. 10.3389/fnsyn.2020.00005 32116641 PMC7026682

[B174] Ramaswamy S. Hill S. L. King J. G. Schurmann F. Wang Y. Markram H. (2012). Intrinsic morphological diversity of thick-tufted layer 5 pyramidal neurons ensures robust and invariant properties of in silico synaptic connections. *J. Physiol.* 590 737–752. 10.1113/jphysiol.2011.219576 22083599 PMC3381307

[B175] Rawson R. L. Martin E. A. Williams M. E. (2017). Mechanisms of input and output synaptic specificity: finding partners, building synapses, and fine-tuning communication. *Curr. Opin. Neurobiol.* 45 39–44. 10.1016/j.conb.2017.03.006 28388510 PMC5554725

[B176] Rees C. L. Moradi K. Ascoli G. A. (2017). Weighing the evidence in Peters’ rule: does neuronal morphology predict connectivity? *Trends Neurosci.* 40 63–71. 10.1016/j.tins.2016.11.007 28041634 PMC5285450

[B177] Reimann M. W. Horlemann A. L. Ramaswamy S. Muller E. B. Markram H. (2017). Morphological diversity strongly constrains synaptic connectivity and plasticity. *Cereb. Cortex* 27 4570–4585. 10.1093/cercor/bhx150 28637203

[B178] Reimann M. W. King J. G. Muller E. B. Ramaswamy S. Markram H. (2015). An algorithm to predict the connectome of neural microcircuits. *Front. Comput. Neurosci.* 9:120. 10.3389/fncom.2015.00120 26500529 PMC4597796

[B179] Reyes A. Lujan R. Rozov A. Burnashev N. Somogyi P. Sakmann B. (1998). Target-cell-specific facilitation and depression in neocortical circuits. *Nat. Neurosci.* 1 279–285. 10.1038/1092 10195160

[B180] Sancho L. Bloodgood B. L. (2018). Functional distinctions between spine and dendritic synapses made onto parvalbumin-positive interneurons in mouse cortex. *Cell Rep.* 24 2075–2087. 10.1016/j.celrep.2018.07.070 30134169

[B181] Sando R. Jiang X. Südhof T. C. (2019). Latrophilin GPCRs direct synapse specificity by coincident binding of FLRTs and teneurins. *Science* 363:eaav7969. 10.1126/science.aav7969 30792275 PMC6636343

[B182] Sando R. Südhof T. C. (2021). Latrophilin GPCR signaling mediates synapse formation. *eLife* 10:e65717. 10.7554/eLife.65717 33646123 PMC7954527

[B183] Sanes J. R. Yamagata M. (2009). Many paths to synaptic specificity. *Annu. Rev. Cell Dev. Biol.* 25 161–195. 10.1146/annurev.cellbio.24.110707.175402 19575668

[B184] Sanes J. R. Zipursky S. L. (2020). Synaptic specificity, recognition molecules, and assembly of neural circuits. *Cell* 181 536–556. 10.1016/j.cell.2020.04.008 32359437

[B185] Scala F. Kobak D. Bernabucci M. Bernaerts Y. Cadwell C. R. Castro J. R. (2020). Phenotypic variation of transcriptomic cell types in mouse motor cortex. *Nature* 10.1038/s41586-020-2907-3 [Epub ahead of print]. 33184512 PMC8113357

[B186] Schafer D. P. Stevens B. (2015). Microglia function in central nervous system development and plasticity. *Cold Spring Harb. Perspect. Biol.* 7:a020545. 10.1101/cshperspect.a020545 26187728 PMC4588063

[B187] Schmidt H. Gour A. Straehle J. Boergens K. M. Brecht M. Helmstaedter M. (2017). Axonal synapse sorting in medial entorhinal cortex. *Nature* 549 469–475. 10.1038/nature24005 28959971

[B188] Schneider-Mizell C. M. Bodor A. L. Collman F. Brittain D. Bleckert A. A. Dorkenwald S. (2020). Chandelier cell anatomy and function reveal a variably distributed but common signal. *bioRxiv* 10.1101/2020.03.31.018952 [Preprint].

[B189] Schroeder A. Vanderlinden J. Vints K. Ribeiro L. F. Vennekens K. M. Gounko N. V. (2018). A modular organization of LRR protein-mediated synaptic adhesion defines synapse identity. *Neuron* 99 329–344.e7. 10.1016/j.neuron.2018.06.026 29983322

[B190] Shapson-Coe A. Januszewski M. Berger D. R. Pope A. Wu Y. E. Blakely T. (2021). A connectomic study of a petascale fragment of human cerebral cortex. *bioRxiv* [Preprint].

[B191] Shen K. Scheiffele P. (2010). Genetics and cell biology of building specific synaptic connectivity. *Annu. Rev. Neurosci.* 33 473–507. 10.1146/annurev.neuro.051508.135302 20367446 PMC3082953

[B192] Shepherd G. M. Stepanyants A. Bureau I. Chklovskii D. Svoboda K. (2005). Geometric and functional organization of cortical circuits. *Nat. Neurosci.* 8 782–790. 10.1038/nn1447 15880111

[B193] Simi A. Studer M. (2018). Developmental genetic programs and activity-dependent mechanisms instruct neocortical area mapping. *Curr. Opin. Neurobiol.* 53 96–102. 10.1016/j.conb.2018.06.007 30005291

[B194] Simon A. Olah S. Molnar G. Szabadics J. Tamas G. (2005). Gap-junctional coupling between neurogliaform cells and various interneuron types in the neocortex. *J. Neurosci.* 25 6278–6285. 10.1523/JNEUROSCI.1431-05.2005 16000617 PMC6725286

[B195] Skutella T. Nitsch R. (2001). New molecules for hippocampal development. *Trends Neurosci.* 24 107–113. 10.1016/s0166-2236(00)01717-3 11164941

[B196] Somogyi P. (1977). A specific ‘axo-axonal’ interneuron in the visual cortex of the rat. *Brain Res.* 136 345–350. 10.1016/0006-8993(77)90808-3 922488

[B197] Somogyi P. (1979). An interneurone making synapses specifically on the axon initial segment of pyramidal cells in the cerebral cortex of the cat [proceedings]. *J. Physiol.* 296 18–19. 529082

[B198] Somogyi P. Freund T. F. Cowey A. (1982). The axo-axonic interneuron in the cerebral cortex of the rat, cat and monkey. *Neuroscience* 7 2577–2607. 10.1016/0306-4522(82)90086-0 7155343

[B199] Song S. Sj̈oström P. J. Reigl M. Nelson S. Chklovskii D. B. (2005). Highly nonrandom features of synaptic connectivity in local cortical circuits. *PLoS Biol.* 3:e68. 10.1371/journal.pbio.0030068 15737062 PMC1054880

[B200] Spence E. F. Dube S. Uezu A. Locke M. Soderblom E. J. Soderling S. H. (2019). In vivo proximity proteomics of nascent synapses reveals a novel regulator of cytoskeleton-mediated synaptic maturation. *Nat. Commun.* 10:386. 10.1038/s41467-019-08288-w 30674877 PMC6344529

[B201] Spruston N. (2008). Pyramidal neurons: dendritic structure and synaptic integration. *Nat. Rev. Neurosci.* 9 206–221. 10.1038/nrn2286 18270515

[B202] Stachniak T. J. Kastli R. Hanley O. Argunsah A. O. Van Der Valk E. G. T. Kanatouris G. (2021). Post-mitotic Prox1 expression controls the final specification of cortical VIP interneuron subtypes. *J. Neurosci.* 10.1523/JNEUROSCI.1021-21.2021 [Epub ahead of print]. 34380763 PMC8482865

[B203] Stachniak T. J. Sylwestrak E. L. Scheiffele P. Hall B. J. Ghosh A. (2019). Elfn1-induced constitutive activation of mGluR7 determines frequency-dependent recruitment of somatostatin interneurons. *J. Neurosci.* 39 4461–4474. 10.1523/JNEUROSCI.2276-18.2019 30940718 PMC6554623

[B204] Staiger J. F. Petersen C. C. H. (2021). Neuronal circuits in barrel cortex for whisker sensory perception. *Physiol. Rev.* 101 353–415. 10.1152/physrev.00019.2019 32816652

[B205] Steinecke A. Hozhabri E. Tapanes S. Ishino Y. Zeng H. Kamasawa N. (2017). Neocortical chandelier cells developmentally shape axonal arbors through reorganization but establish subcellular synapse specificity without refinement. *eNeuro* 4:ENEURO.0057-17.2017. 10.1523/ENEURO.0057-17.2017 28584877 PMC5458751

[B206] Stepanyants A. Chklovskii D. B. (2005). Neurogeometry and potential synaptic connectivity. *Trends Neurosci.* 28 387–394. 10.1016/j.tins.2005.05.006 15935485

[B207] Stepanyants A. Hirsch J. A. Martinez L. M. Kisvarday Z. F. Ferecsko A. S. Chklovskii D. B. (2008). Local potential connectivity in cat primary visual cortex. *Cereb. Cortex* 18 13–28. 10.1093/cercor/bhm027 17420172

[B208] Stepanyants A. Tamas G. Chklovskii D. B. (2004). Class-specific features of neuronal wiring. *Neuron* 43 251–259. 10.1016/j.neuron.2004.06.013 15260960

[B209] Stogsdill J. A. Eroglu C. (2017). The interplay between neurons and glia in synapse development and plasticity. *Curr. Opin. Neurobiol.* 42 1–8. 10.1016/j.conb.2016.09.016 27788368 PMC5316301

[B210] Stratford K. J. Tarczy-Hornoch K. Martin K. A. Bannister N. J. Jack J. J. (1996). Excitatory synaptic inputs to spiny stellate cells in cat visual cortex. *Nature* 382 258–261. 10.1038/382258a0 8717041

[B211] Stuart G. J. Spruston N. (2015). Dendritic integration: 60 years of progress. *Nat. Neurosci.* 18 1713–1721. 10.1038/nn.4157 26605882

[B212] Südhof T. C. (2018). Towards an understanding of synapse formation. *Neuron* 100 276–293. 10.1016/j.neuron.2018.09.040 30359597 PMC6226307

[B213] Sun Y. C. Chen X. Fischer S. Lu S. Zhan H. Gillis J. (2021). Integrating barcoded neuroanatomy with spatial transcriptional profiling enables identification of gene correlates of projections. *Nat. Neurosci.* 24 873–885. 10.1038/s41593-021-00842-4 33972801 PMC8178227

[B214] Suto F. Tsuboi M. Kamiya H. Mizuno H. Kiyama Y. Komai S. (2007). Interactions between plexin-A2, plexin-A4, and semaphorin 6A control lamina-restricted projection of hippocampal mossy fibers. *Neuron* 53 535–547. 10.1016/j.neuron.2007.01.028 17296555

[B215] Sylwestrak E. L. Ghosh A. (2012). Elfn1 regulates target-specific release probability at CA1-interneuron synapses. *Science* 338 536–540. 10.1126/science.1222482 23042292 PMC5297939

[B216] Tai Y. Gallo N. B. Wang M. Yu J. R. Van Aelst L. (2019). Axo-axonic innervation of neocortical pyramidal neurons by GABAergic Chandelier cells requires AnkyrinG-associated L1CAM. *Neuron* 102 358–372.e9. 10.1016/j.neuron.2019.02.009 30846310 PMC6525570

[B217] Tai Y. Janas J. A. Wang C. L. Van Aelst L. (2014). Regulation of chandelier cell cartridge and bouton development via DOCK7-mediated ErbB4 activation. *Cell Rep.* 6 254–263. 10.1016/j.celrep.2013.12.034 24440718 PMC3920736

[B218] Tan Z. Hu H. Huang Z. J. Agmon A. (2008). Robust but delayed thalamocortical activation of dendritic-targeting inhibitory interneurons. *Proc. Natl. Acad. Sci. U. S. A.* 105 2187–2192. 10.1073/pnas.0710628105 18245383 PMC2538896

[B219] Tarusawa E. Sanbo M. Okayama A. Miyashita T. Kitsukawa T. Hirayama T. (2016). Establishment of high reciprocal connectivity between clonal cortical neurons is regulated by the Dnmt3b DNA methyltransferase and clustered protocadherins. *BMC Biol.* 14:103. 10.1186/s12915-016-0326-6 27912755 PMC5133762

[B220] Tasic B. Menon V. Nguyen T. N. Kim T. K. Jarsky T. Yao Z. (2016). Adult mouse cortical cell taxonomy revealed by single cell transcriptomics. *Nat. Neurosci.* 19 335–346. 10.1038/nn.4216 26727548 PMC4985242

[B221] Tasic B. Yao Z. Graybuck L. T. Smith K. A. Nguyen T. N. Bertagnolli D. (2018). Shared and distinct transcriptomic cell types across neocortical areas. *Nature* 563 72–78. 10.1038/s41586-018-0654-5 30382198 PMC6456269

[B222] Thomson A. M. Lamy C. (2007). Functional maps of neocortical local circuitry. *Front. Neurosci.* 1:19–42. 10.3389/neuro.01.1.1.002.2007 18982117 PMC2518047

[B223] Tomioka N. H. Yasuda H. Miyamoto H. Hatayama M. Morimura N. Matsumoto Y. (2014). Elfn1 recruits presynaptic mGluR7 in trans and its loss results in seizures. *Nat. Commun.* 5:4501. 10.1038/ncomms5501 25047565

[B224] Tran T. S. Rubio M. E. Clem R. L. Johnson D. Case L. Tessier-Lavigne M. (2009). Secreted semaphorins control spine distribution and morphogenesis in the postnatal CNS. *Nature* 462 1065–1069. 10.1038/nature08628 20010807 PMC2842559

[B225] Tremblay R. Lee S. Rudy B. (2016). GABAergic interneurons in the neocortex: from cellular properties to circuits. *Neuron* 91 260–292. 10.1016/j.neuron.2016.06.033 27477017 PMC4980915

[B226] Udvary D. Harth P. Macke J. H. Hege H.-C. De Kock C. P. Sakmann B. (2021). The impact of neuronal structure on cortical network architecture. *bioRxiv* 10.1101/2020.11.13.381087 [Preprint]. PMC9035680 35417720

[B227] Varga C. Lee S. Y. Soltesz I. (2010). Target-selective GABAergic control of entorhinal cortex output. *Nat. Neurosci.* 13 822–824. 10.1038/nn.2570 20512133 PMC3139425

[B228] Wang X. Sun Q. Q. (2012). Characterization of axo-axonic synapses in the piriform cortex of Mus musculus. *J. Comp. Neurol.* 520 832–847. 10.1002/cne.22792 22020781 PMC3903392

[B229] Wang X. Tucciarone J. Jiang S. Yin F. Wang B. S. Wang D. (2019). Genetic single neuron anatomy reveals fine granularity of cortical axo-axonic cells. *Cell Rep.* 26 3145–3159.e5. 10.1016/j.celrep.2019.02.040 30865900 PMC7863572

[B230] Wang Y. Gupta A. Toledo-Rodriguez M. Wu C. Z. Markram H. (2002). Anatomical, physiological, molecular and circuit properties of nest basket cells in the developing somatosensory cortex. *Cereb. Cortex* 12 395–410. 10.1093/cercor/12.4.395 11884355

[B231] Wang Y. Markram H. Goodman P. H. Berger T. K. Ma J. Goldman-Rakic P. S. (2006). Heterogeneity in the pyramidal network of the medial prefrontal cortex. *Nat. Neurosci.* 9 534–542. 10.1038/nn1670 16547512

[B232] Wefelmeyer W. Cattaert D. Burrone J. (2015). Activity-dependent mismatch between axo-axonic synapses and the axon initial segment controls neuronal output. *Proc. Natl. Acad. Sci. U. S. A.* 112 9757–9762. 10.1073/pnas.1502902112 26195803 PMC4534224

[B233] Wen Q. Stepanyants A. Elston G. N. Grosberg A. Y. Chklovskii D. B. (2009). Maximization of the connectivity repertoire as a statistical principle governing the shapes of dendritic arbors. *Proc. Natl. Acad. Sci. U. S. A.* 106 12536–12541. 10.1073/pnas.0901530106 19622738 PMC2713752

[B234] West D. C. Mercer A. Kirchhecker S. Morris O. T. Thomson A. M. (2006). Layer 6 cortico-thalamic pyramidal cells preferentially innervate interneurons and generate facilitating EPSPs. *Cereb. Cortex* 16 200–211. 10.1093/cercor/bhi098 15843627

[B235] Wu P. R. Cho K. K. A. Vogt D. Sohal V. S. Rubenstein J. L. R. (2017). The cytokine CXCL12 promotes basket interneuron inhibitory synapses in the medial prefrontal cortex. *Cereb. Cortex* 27 4303–4313. 10.1093/cercor/bhw230 27497284 PMC6410508

[B236] Xie Y. Hkuan A. T. Wang W. Herbert Z. T. Mosto O. Olukoya O. (2020). Astrocyte-neuron crosstalk through Hedgehog signaling mediates cortical circuit assembly. *bioRxiv* [Preprint]. 10.1016/j.celrep.2022.110416 PMC8962654 35196485

[B237] Xu H. Jeong H. Y. Tremblay R. Rudy B. (2013). Neocortical somatostatin-expressing GABAergic interneurons disinhibit the thalamorecipient layer 4. *Neuron* 77 155–167. 10.1016/j.neuron.2012.11.004 23312523 PMC3556168

[B238] Yang J. M. Shen C. J. Chen X. J. Kong Y. Liu Y. S. Li X. W. (2019). *erbb4* deficits in Chandelier cells of the medial prefrontal cortex confer cognitive dysfunctions: implications for schizophrenia. *Cereb. Cortex* 29 4334–4346. 10.1093/cercor/bhy316 30590426

[B239] Yao Z. Liu H. Xie F. Fischer S. Booeshaghi A. S. Adkins R. S. (2020). An integrated transcriptomic and epigenomic atlas of mouse primary motor cortex cell types. *bioRxiv* 10.1101/2020.02.29.970558 [Preprint].

[B240] Yassin L. Benedetti B. L. Jouhanneau J. S. Wen J. A. Poulet J. F. Barth A. L. (2010). An embedded subnetwork of highly active neurons in the neocortex. *Neuron* 68 1043–1050. 10.1016/j.neuron.2010.11.029 21172607 PMC3022325

[B241] Yu Y. C. Bultje R. S. Wang X. Shi S. H. (2009). Specific synapses develop preferentially among sister excitatory neurons in the neocortex. *Nature* 458 501–504. 10.1038/nature07722 19204731 PMC2727717

[B242] Yu Y. C. He S. Chen S. Fu Y. Brown K. N. Yao X. H. (2012). Preferential electrical coupling regulates neocortical lineage-dependent microcircuit assembly. *Nature* 486 113–117. 10.1038/nature10958 22678291 PMC3599787

[B243] Yuste R. (2011). Dendritic spines and distributed circuits. *Neuron* 71 772–781. 10.1016/j.neuron.2011.07.024 21903072 PMC4071954

[B244] Yuste R. Hawrylycz M. Aalling N. Aguilar-Valles A. Arendt D. Armananzas R. (2020). A community-based transcriptomics classification and nomenclature of neocortical cell types. *Nat. Neurosci.* 23 1456–1468. 10.1038/s41593-020-0685-8 32839617 PMC7683348

[B245] Zeisel A. Munoz-Manchado A. B. Codeluppi S. Lonnerberg P. La Manno G. Jureus A. (2015). Brain structure. Cell types in the mouse cortex and hippocampus revealed by single-cell RNA-seq. *Science* 347 1138–1142. 10.1126/science.aaa1934 25700174

[B246] Zeng H. Sanes J. R. (2017). Neuronal cell-type classification: challenges, opportunities and the path forward. *Nat. Rev. Neurosci.* 18 530–546. 10.1038/nrn.2017.85 28775344

[B247] Zhang X. J. Li Z. Han Z. Sultan K. T. Huang K. Shi S. H. (2017). Precise inhibitory microcircuit assembly of developmentally related neocortical interneurons in clusters. *Nat. Commun.* 8:16091. 10.1038/ncomms16091 28703129 PMC5511369

[B248] Ziv N. E. Smith S. J. (1996). Evidence for a role of dendritic filopodia in synaptogenesis and spine formation. *Neuron* 17 91–102. 10.1016/s0896-6273(00)80283-4 8755481

